# Dual-Functional Schottky-Barrier-Free Plasmonic TiN/TiO_2_ Photocatalyst for Efficient NH_3_ and H_2_ Production

**DOI:** 10.1021/acsami.5c06863

**Published:** 2025-06-13

**Authors:** Xiaopeng Bai, Ke An, Lingyu Jia, Yanzhen Guo, Jingtian Hu, Guangri Jia, Ruibin Jiang, Jianfang Wang, Jimmy C. Yu

**Affiliations:** † Department of Physics, 26451 The Chinese University of Hong Kong, Shatin, Hong Kong SAR 999077, China; ‡ Shaanxi Key Laboratory for Advanced Energy Devices, Shaanxi Engineering Lab for Advanced Energy Technology, School of Materials Science and Engineering, 12401 Shaanxi Normal University, Xi’an, Shaanxi 710119, China; § Department of Chemistry, The Chinese University of Hong Kong, Shatin, Hong Kong SAR 999077, China; ∥ Henan Provincial Key Laboratory of Nanocomposites and Applications, Institute of Nanostructured Functional Materials, 118305 Huanghe Science and Technology College, Zhengzhou, Henan 450006, China

**Keywords:** nitrogen photofixation, photocatalytic hydrogen production, plasmonic photocatalysts, titania, titanium nitride

## Abstract

Titanium nitride has garnered much attention owing to its high charge carrier density and low work function. These merits compensate for the shortcomings of traditional photocatalysts. Herein we report on the synthesis of TiN/TiO_2_ nanoparticles (NPs) as a new Schottky-barrier-free plasmonic photocatalyst (SBFPP) through the oxidation of commercially available TiN NPs. Our SBFPP possesses abundant oxygen vacancies (OVs) and the ability to generate hot charge carriers. The TiN NPs oxidized at 400 °C for 2 h, which is designated as TiN-2, exhibit broad light absorption and a high OV concentration. Theoretical calculations have revealed that the presence of OVs and nitrogen dopants leads to the formation of defect electronic states in TiO_2_. Hot electrons from TiN can efficiently migrate to these defect states, hindering the rapid electron–hole recombination. Besides the efficient photocatalytic nitrogen fixation, the TiN-2 NPs also exhibit excellent hydrogen generation performance. Furthermore, the photocatalytic film formed by combining the TiN/TiO_2_ NPs and a porous poly­(vinyl alcohol) film dramatically improves the photocatalytic nitrogen fixation performance, giving an enhancement of ∼4.4 times in comparison with the TiN-2 NPs. This study unveils a promising avenue for the rational design of plasmonic photocatalysts and facilitates the development of practical applications in the field of photocatalysis.

## Graphical abstract

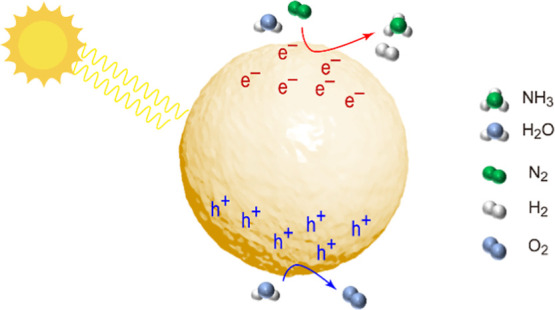

## 1 Introduction

The Haber-Bosch process represents a well-established industrial nitrogen fixation technology that supplies nearly a half of the nitrogen source on the world.^1^ This process involves the synthesis of ammonia through the coactivation of nitrogen and hydrogen molecules on iron-based catalysts at high temperatures (>300 °C) and pressures (>100 atm).^2^ A significant challenge for this process is the overcoming of the robust triple N≡N bond with a bond dissociation enthalpy of 941 kJ mol^–1^. As a result, industrial ammonia production consumes over 1.4% of the global energy supply and contributes approximately 1.6% of the global CO_2_ emissions.^3^

Plasmonically powered photocatalytic nitrogen fixation has emerged as a promising alternative to reduce energy consumption and greenhouse gas emissions. This approach involves the utilization of the plasmonic properties of nanomaterials, whose localized surface plasmon resonance can efficiently facilitate the photogeneration of hot charge carriers. There are three types of plasmonic photocatalysts: plasmonic metals, metal–semiconductor hybrid structures, and Schottky-barrier-free plasmonic materials.^4^ Metallic plasmonic photocatalysts are usually composed of noble metals and catalytically active metals, which possess strong light absorption over a wide adjustable range. However, this type of plasmonic photocatalyst has limited efficiencies because of the rapid recombination of plasmonically generated charge carriers.^5,6^ The hybridization of plasmonic metals with semiconductors is an effective strategy to increase the lifetime of plasmonically generated charge carriers. The metal–semiconductor junction can greatly improve the separation efficiency of plasmonic hot charge carriers and thus vastly enhance their utilization efficiency.^7–9^ However, plasmonic hot carriers produced by nonradiative decay need to be extracted very rapidly (within ∼ 1 ps), and the existence of the Schottky barrier reduces the transfer efficiency of plasmonic hot charge carriers from the metal to the semiconductor. Many hot charge carriers with insufficient energies cannot overcome the Schottky barrier and hence are lost in the plasmonic metal eventually.^10^

We have proposed and developed Schottky-barrier-free plasmonic photocatalysts (SBFPPs) to overcome this problem.^11^ SBFPPs enable the direct transfer of hot charge carriers to active sites and can significantly enhance the utilization of hot carriers for catalytic reactions. Our previous work has demonstrated molybdenum oxide (MoO_3–*x*_) with abundant oxygen vacancies (OVs) as a potential SBFPP for nitrogen fixation.^11^ However, SBFPPs have so far been limited to only a few materials, which significantly restricts the improvement of the photocatalytic efficiency. To attack this issue, we reason that plasmonic materials with low work functions can probably function as SBFPPs. Recent studies have shown that TiN is a promising alternative plasmonic material to replace noble metals.^12,13^ TiN possesses a high carrier density that is comparable to those in noble metals and thus exhibits strong visible and near-infrared (NIR) light absorption.^14,15^ Furthermore, the low work function of TiN facilitates the transfer of photoexcited hot electrons to adjacent species, increasing their likelihood of participating in catalytic reactions at active sites.^16^ Up to now, TiN nanoparticles (NPs) have been used as photocatalysts for various reactions such as the photocatalytic production of formate,^17^ hydroalumination,^18^ and CO_2_ reduction,^19^ but it has not been employed for nitrogen fixation.

In this work, TiN/TiO_2_ NPs were designed as a new type of SBFPP. They are synthesized simply by adjusting the oxidation extent of commercially available TiN NPs and named as TiN-*x*, with *x* representing the oxidation time in hour. The synthesized TiN-*x* NPs exhibit strong light absorption across the ultraviolet, visible, and NIR regions. Broadband light absorption enables the TiN-*x* NPs to harness solar energy efficiently, thereby enhancing their photocatalytic nitrogen fixation efficiency. Notably, TiN-2 NPs displayed a higher concentration of OVs than the other samples, which benefits hot charge carrier utilization and N_2_ activation. Density functional theory (DFT) calculations revealed the presence of defect electronic states between the conduction band (CB) and valence band (VB) of the TiO_2_ component in the TiN-2 NPs owing to the presence of OVs and N dopants. The states are situated below the Fermi level of TiN. Consequently, hot electrons can efficiently transfer from TiN to the CB of TiO_2_ and then to the defect states without encountering any Schottky barrier.^20^ Moreover, the defect states can capture and trap charge carriers, preventing or delaying their recombination. This trapping effect increases the lifetime of charge carriers, endowing them with a high probability of participating in photocatalytic reactions.^21^ The distinct structural advantages of the TiN/TiO_2_ NPs, especially the TiN-2 NPs, display notable photoactivities in NH_3_ generation. The apparent quantum efficiency (AQE) of the TiN-2 NPs surpasses 0.08% at 700 nm without using any hole scavenger. The solar-to-chemical conversion efficiency (SCCE) surpasses 0.02%. In addition to photocatalytic nitrogen fixation, the TiN-2 NPs exhibit impressive hydrogen and oxygen generation activities. Given their multifunctional photocatalytic activities, straightforward preparation process, and efficient utilization of hot electrons, the TiN/TiO_2_ NPs as a new SBFPP offer inspiration for the further design of other innovative SBFPPs.

## 2 Results and Discussion

The TiN/TiO_2_ NPs were synthesized through the oxidation of commercially available TiN NPs in ambient air. To find the appropriate temperature for the transformation from TiN to TiO_2_, thermogravimetric analysis (TGA) was performed. The result reveals an inflection point at ∼68 min (Figure S1a), after which the weight loss ceases. Given the heating rate of 5 °C min^–1^ with a 30 °C initial temperature, the inflection point corresponds to roughly 370 °C (Figure S1b). This observation indicates that all adsorbed molecules, such as water and carbon species in air, are completely removed at this temperature.^22^ We therefore reasoned that when the temperature is set at 400 °C, TiN can be transformed into TiO_2_ at a slow rate while adsorbed species can be removed. To effectively control the transformation extent of TiN to TiO_2_, a geometric sequence of the duration of oxidation at 400 °C was employed to yield a series of samples (Figure 1a). As the holding time of the TiN NPs was increased, significant changes in color and composition were observed. The samples vary from black to gray and finally to light yellow within 16 h (Figure S2). Their sizes and morphologies were characterized by scanning electron microscopy (SEM, Figure S3) and transmission electron microscopy (TEM) (Figure S4). The average sizes of the particles remain constant at 20 nm, indicating that the heat treatment has a negligible effect on the size of the TiN NPs. The TEM images reveal a clustered network structure formed by the TiN NPs, which is caused by the van der Waals forces among the NPs^23^ and the thermal treatment. Moreover, the Brunauer–Emmett–Teller (BET) analysis revealed that the specific surface areas of the TiN-0, TiN-2, and TiN-16 NPs are 44.3 m^2^ g^–1^, 40.6 m^2^ g^–1^, and 39.1 m^2^ g^–1^, respectively (Figure S5), confirming that the TiN-*x* samples have comparable specific surface areas. This observation is consistent with their similar particle sizes and morphologies (Figures S3 and S4).

**Figure 1:**
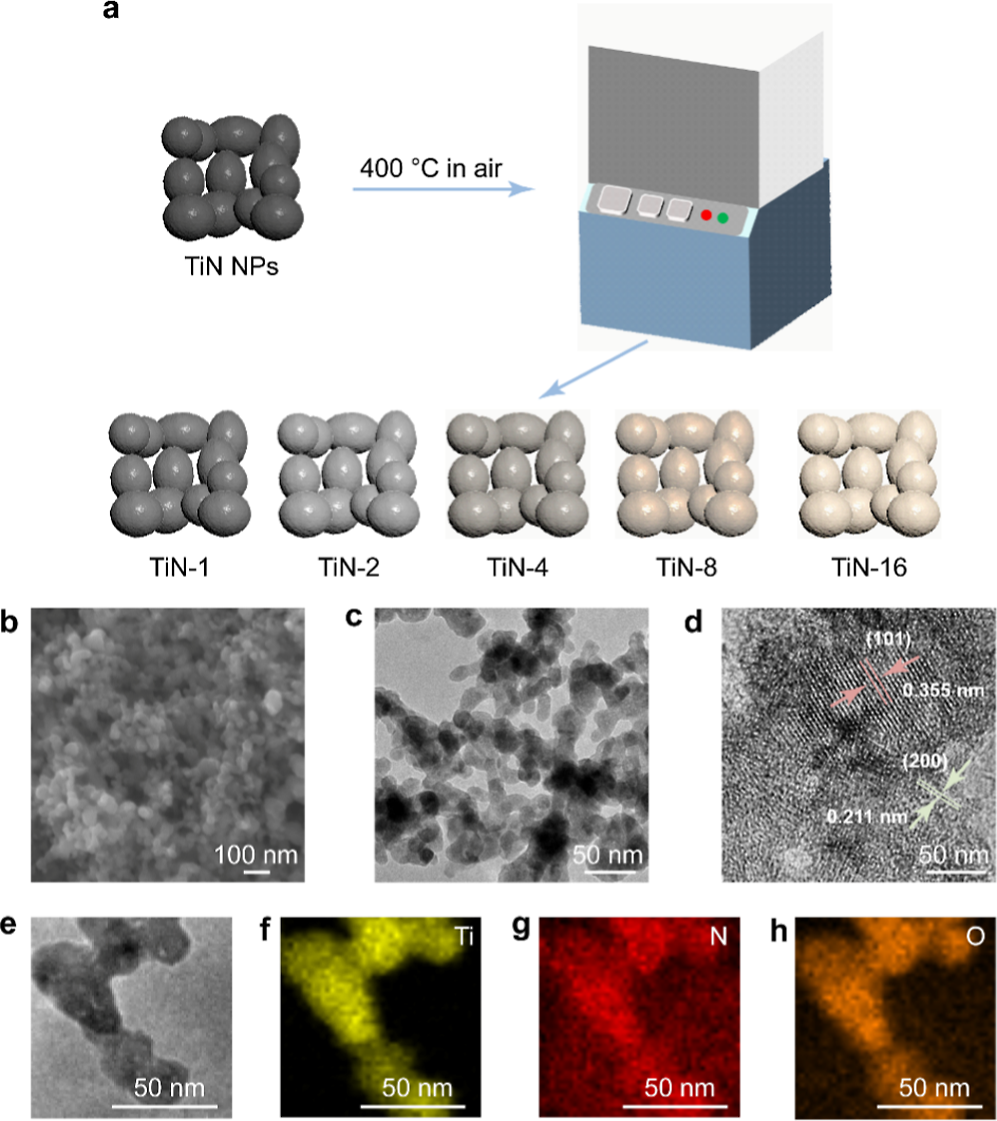
Schematics and characterization of the TiN-*x* NPs. (a) Schematics illustrating the preparation process of the TiN-*x* NPs. (b) SEM image of the TiN-2 NPs. (c) TEM image of the TiN-2 NPs. (d) HRTEM image of the TiN-2 NPs. (e) TEM image of the TiN-2 NPs. (f–h) EDX mapping of the TiN-2 NPs.

The SEM and TEM images (Figure 1b,c) show that the morphology of the TiN-2 NPs is similar to those of the other samples. The high-resolution TEM (HRTEM) images reveal changes in the lattice spacing of the TiN NPs after oxidation (Figure 1d). A lattice spacing of 0.211 nm was observed, which is in agreement with the TiN (200) lattice planes with a spacing of 0.212 nm. In addition, lattice planes with a spacing of 0.355 nm was also identified, indicating the existence of the anatase TiO_2_ (101) lattice planes (0.352 nm). The distinct and well-defined crystalline structure attests to the high crystallinity of the TiN-2 NPs. The oxidation process likely results in a gradient of TiO_2_ formation from the surface inward, creating a heterojunction-like interface between TiN and TiO_2_. This interface facilitates efficient transfer of hot electrons from TiN to TiO_2_. It is noteworthy that the anatase-type TiO_2_ typically exhibits a higher density of lattice defects than the rutile-type TiO_2_, which can potentially enhance the catalytic performance through the excitation of electrons from the VB to the defect states.^24^ Energy-dispersive X-ray (EDX) mapping confirmed the presence of the Ti, O, and N elements in the TiN-2 NPs, indicating the coexistence of TiN and TiO_2_ rather than a core@shell structure (Figure 1e–h). This structural arrangement enhances the utilization of photogenerated charge carriers in the TiN component for catalysis, thus promoting the photocatalytic activity.

The X-ray diffraction (XRD) pattern of the original TiN NPs is consistent with the standard one of hexagonal TiN (JCPDS no. 87-0628). TiN and TiO_2_ coexisted in the sample when the holding time was shorter than 2 h. However, as the holding time surpassed 2 h, TiO_2_ became the dominant phase, and the TiN content decreased significantly (Figure 2a). For the TiN-4 sample, the XRD pattern (Figure 2a) shows peaks arising from both TiN and TiO_2_, indicating that while a portion of TiN has been oxidized to TiO_2_, a noticeable portion of TiN remains stable under thermal treatment at 400 °C for 4 h. In addition, the XRD measurements indicated that the rutile phase is present only in trace amounts in our TiN-*x* samples. This is consistent with the fact that the anatase-to-rutile phase transition in pure TiO_2_ typically occurs between 600 and 700 °C.^25^ As a result, the overall phase compositions of our TiN-*x* samples remain to be dominated by the anatase phase, which is known to have superior photocatalytic performances compared to the rutile phase. The Fourier-transform infrared (FT-IR) spectra reveal that with increasing oxidation time, the infrared signal corresponding to the Ti–O–H bonds gradually strengthens, while the signal associated with the Ti–N bonds decreases in intensity (Figure 2b). The X-ray photoelectron spectroscopy (XPS) spectra of the TiN NPs confirm the presence of a tiny amount of TiO_2_ owing to the formation of a native 1–2 nm thick TiO_2_ oxide layer on the TiN surface (Figure 2c).^26–28^ The XPS spectra of the TiN-*x* NPs indicate that as the heating duration was lengthened, TiN was progressively oxidized, resulting in gradual increases in the TiO_2_ content.^29^ For the O 1s spectra, the concentration of the Ti–O bonds increases with extended heating durations, while the concentration of the O–N bonds decreases (Figure S6). This result indicates that the surface nitrogen atoms are gradually depleted with increasing oxidation durations. EDX analysis indicates that the N content in the TiN-*x* samples gradually decreases with increasing holding time, while the oxygen content shows an increasing trend (Figure S7).

**Figure 2:**
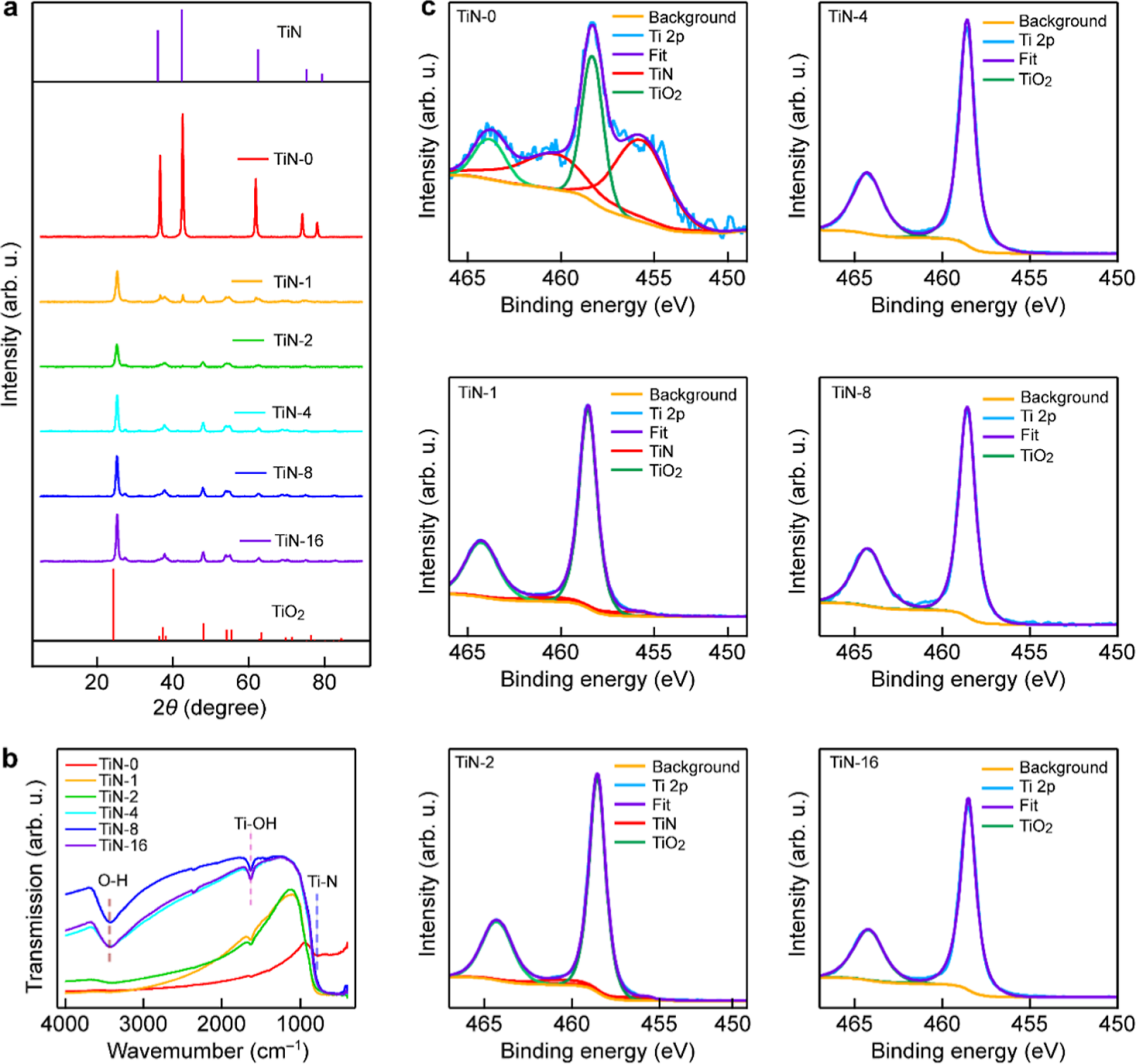
Characterization of the TiN-*x* NPs. (a) XRD patterns of the TiN NPs treated for different holding durations. (b) FT-IR spectra of the TiN-*x* NPs. (c) XPS spectra of the TiN-*x* NPs.

The zeta potential test results indicate that both of the TiN-0 and TiN-2 NPs possess negative potentials at −0.30 mV and −0.36 mV, respectively (Figure S8). Such zeta potentials are relatively small, implying that their surfaces carry a small number of negative charges. The negative charges are insufficient for preventing the NPs from aggregation and flocculation, as observed in our experiments. Since the NP sizes are roughly 20 nm, aggregation among the NPs causes plasmon coupling, which in turn generates abundant hot spots on the NP surface. The hot spots amplify the local electromagnetic field, thereby increasing light absorption in these regions.^30^ This enhanced light absorption increases the generation of electron–hole pairs in the photocatalyst, which is a crucial step in the photocatalytic process. In addition, the generation of electron–hole pairs in the photocatalyst is beneficial only when photogenerated charge carriers (electrons and holes) are efficiently separated. The localized field enhancement can promote specific chemical reactions by lowering their activation energy barriers.^31^

The absorption spectra of the TiN NPs in the visible range display a declining trend as the holding time for the thermal treatment is increased (Figure 3a). This phenomenon is attributed to the increasing TiO_2_ composition within the sample as the holding time is increased because TiO_2_ primarily absorbs ultraviolet light. Compared to the mixed sample of commercial TiN and TiO_2_, the TiN-2 sample exhibits stronger light absorption (Figure S9). This suggests that the TiN and TiO_2_ components in the TiN-2 NPs have interactions that can enhance light absorption. Furthermore, the bandgap of the TiN-*x* NPs can be estimated from their respective absorption spectra (Figure S10).^32^ The flatband positions of the TiN-*x* NPs were determined from the Mott–Schottky tests (Figure 3b). The band structures of the TiN-*x* NPs were deduced by combining the flatband and diffuse reflectance spectroscopy (DRS) results (Table S1). The work functions of the TiN NPs were determined from ultraviolet photoelectron spectroscopy (UPS, Figure 3c). They provide insight into the direction of electron transfer. The low work function of TiN enables its Fermi level to be higher than the CB and defect states of TiO_2_. Consequently, hot electrons generated in TiN can readily migrate to active sites on the TiO_2_ component in the TiN-2 NPs. Mott–Schottky measurements also confirmed the high carrier concentration of the TiN NPs (Figure S11). The carrier concentration of the TiN NPs was determined to be 3.02 × 10^22^ cm^–3^ through linear fitting along the linear segment of the Mott–Schottky plot. It is comparable to those of noble metals.^33^ The charge carrier concentrations of the TiN-*x* NPs are shown in Figure 3d. The high carrier concentration of the TiN NPs suggests that they possess plasmonic characteristics, enabling them to produce a number of hot electrons under light excitation.

**Figure 3:**
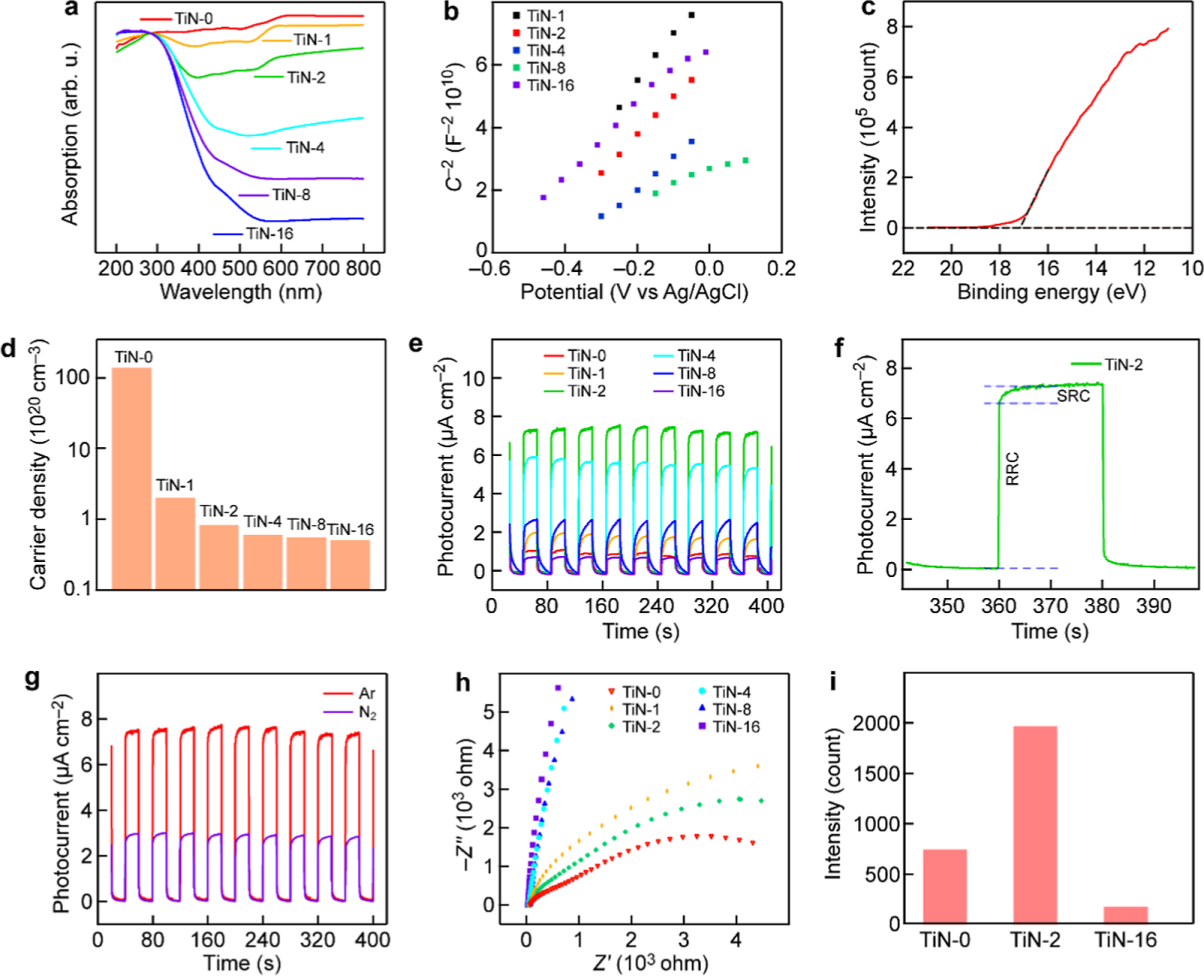
Optical and electrical properties of the TiN-*x* NPs. (a) Absorption spectra of the TiN-*x* NPs. (b) Mott–Schottky plots of the TiN-*x* NPs measured at the frequency of 1000 Hz. (c) UPS spectrum of the TiN NPs. (d) Charge carrier densities of the TiN-*x* NPs. (e) Photocurrent responses of the TiN-*x* NPs. (f) Photocurrent response of the TiN-2 NPs. (g) Photocurrent response of the TiN-2 NPs in two different gas atmospheres. (h) EIS spectra of the TiN-*x* NPs. (i) EPR spectra of the TiN-*x* NPs.

The photocurrent responses of the TiN-*x* NPs were systematically examined using an electrochemical workstation to investigate the behavior of photogenerated electrons within the TiN-*x* NPs (Figure 3e). The response stability of the photocurrent density of the samples was evaluated by cycling the light source on and off at 20 s intervals. Among all the samples, the TiN-1 NPs have the smallest amount of TiO_2_. Their relatively low photocurrent can be attributed to the fact that the small amount of TiO_2_ in the sample leads to the rapid recombination of photogenerated electrons and holes. The TiN-2 NPs exhibit the highest photocurrent density and excellent photocurrent response stability, showing that they have the most effective charge generation and transfer capability.^34^ Moreover, the photocurrent curve of the TiN-2 NPs displays two distinct regions: the rapid response current (RRC) region and the slow response current (SRC) region (Figure 3f). The RRC region is associated with the photoelectric effect of the localized plasmon resonance, while the SRC region arises from the photothermal effect of the plasmon resonance. The shark-fin-like shape of the photocurrent signal results from the capture of photogenerated electrons by OVs in the sample. This phenomenon is similar to the inductive effect and results from the delayed generation and attenuation of the photocurrent signal.^35^ The photocurrent signals in argon and nitrogen atmospheres were also measured (Figures 3g, S12 and S13). Among the different samples, the TiN-2 NPs exhibit the most substantial reduction in photocurrent. In the argon atmosphere, the photocurrent signal of the TiN-2 NPs reaches 7.76 μA cm^–2^, while in the nitrogen atmosphere, the photocurrent signal of the TiN-2 NPs decreases to 4.75 μA cm^–2^. The reduced photocurrent is attributed to the consumption of photogenerated electrons during the photocatalytic nitrogen fixation reaction. The high photocurrent reduction confirms that the TiN-2 NPs enable more efficient utilization of photogenerated electrons in photocatalytic nitrogen fixation than the other samples (Table S2). Furthermore, the smaller semicircles in the electrochemical impedance spectroscopy (EIS) results of the TiN-*x* NPs (Figure 3h) indicate enhanced charge transfer and faster surface reaction kinetics.^36^ The TiN-2 NPs show the highest charge separation efficiency, which is reflected by the smallest arc radius among all the samples.^37^ To confirm the presence of OVs in the sample, electron paramagnetic resonance (EPR) spectroscopy was employed to detect the existence of unpaired electrons. The results reveal a substantial presence of OVs in the TiN-2 NPs, with the EPR signal strength being 11.1 times stronger than that observed for the TiN-16 NPs (Figures 3i and S14). The EPR signal intensity is a semiquantitative measure of the OV concentration. The 11.1 times stronger EPR signal of TiN-2 than that of TiN-16 implies a significantly higher density of OVs in TiN-2. In addition, EDX analysis shows that TiN-2 retains approximately 10 at % nitrogen, suggesting substantial nitrogen doping in the TiO_2_ lattice (Figure S7). The superior photocatalytic performance of TiN-2 correlates with its high OV concentration and high nitrogen doping level, highlighting the importance of these defects in enhancing charge separation and transfer.

The photocatalytic nitrogen fixation tests were performed with a homemade setup (Figure S15). A linear relationship between the light absorbance and the ammonium concentration was established using a chromogenic method to determine the concentration of produced ammonium ions (Figure S16). The concentration of ammonium ions in solution was determined by measuring the absorbance at 425 nm. The photocatalytic nitrogen fixation performance of the TiN-*x* NPs is shown in Figure 4a,b. The TiN-2 NPs exhibit the best photocatalytic nitrogen fixation performance, with an ammonia production rate of 136.4 μmol g^–1^ h^–1^. To verify that both nitrogen and light are essential in the photocatalytic nitrogen fixation process, photocatalytic nitrogen fixation with the TiN-2 NPs was tested under different conditions, which included nitrogen with light, nitrogen without light, and argon with light (Figure S17). The results reveal that ammonia was only produced under the conditions of nitrogen with light, while negligible amounts of ammonia were generated under the other conditions. This finding confirms that light is the driving force for photocatalytic nitrogen fixation and that N_2_ serves as the nitrogen source to produce ammonium ions. In addition, the ^15^N_2_ isotope labeling experiments were performed with the TiN-2 NPs. As displayed in Figure S18, the doublet peaks of ^15^NH_4_^+^ were observed in the ^1^H nuclear magnetic resonance spectra, which further proved that the detected ammonia was generated from nitrogen gas. Moreover, the TiN-2 NP photocatalyst displays favorable photocatalytic stability (Figure 4c). The photocatalytic nitrogen fixation activity remained consistent after 10 cycles of the photocatalytic test. SEM imaging showed that the sample before and after the reaction remained nearly unchanged (Figure S19), indicating that the photocatalytic nitrogen fixation reaction has little impact on the surface morphology of the photocatalyst sample. In addition, the XRD patterns and infrared spectra of the TiN-2 NPs before and after the photocatalytic nitrogen fixation reaction remained nearly identical (Figure S20). Furthermore, the XPS spectra of Ti 2p and O 1s recorded before and after the photocatalytic reaction confirmed that the composition of the photocatalyst remained nearly unchanged during the reaction (Figure S21). All of these results show that the composition and structure of the TiN-2 NPs are preserved throughout the photocatalytic nitrogen fixation reaction.

**Figure 4:**
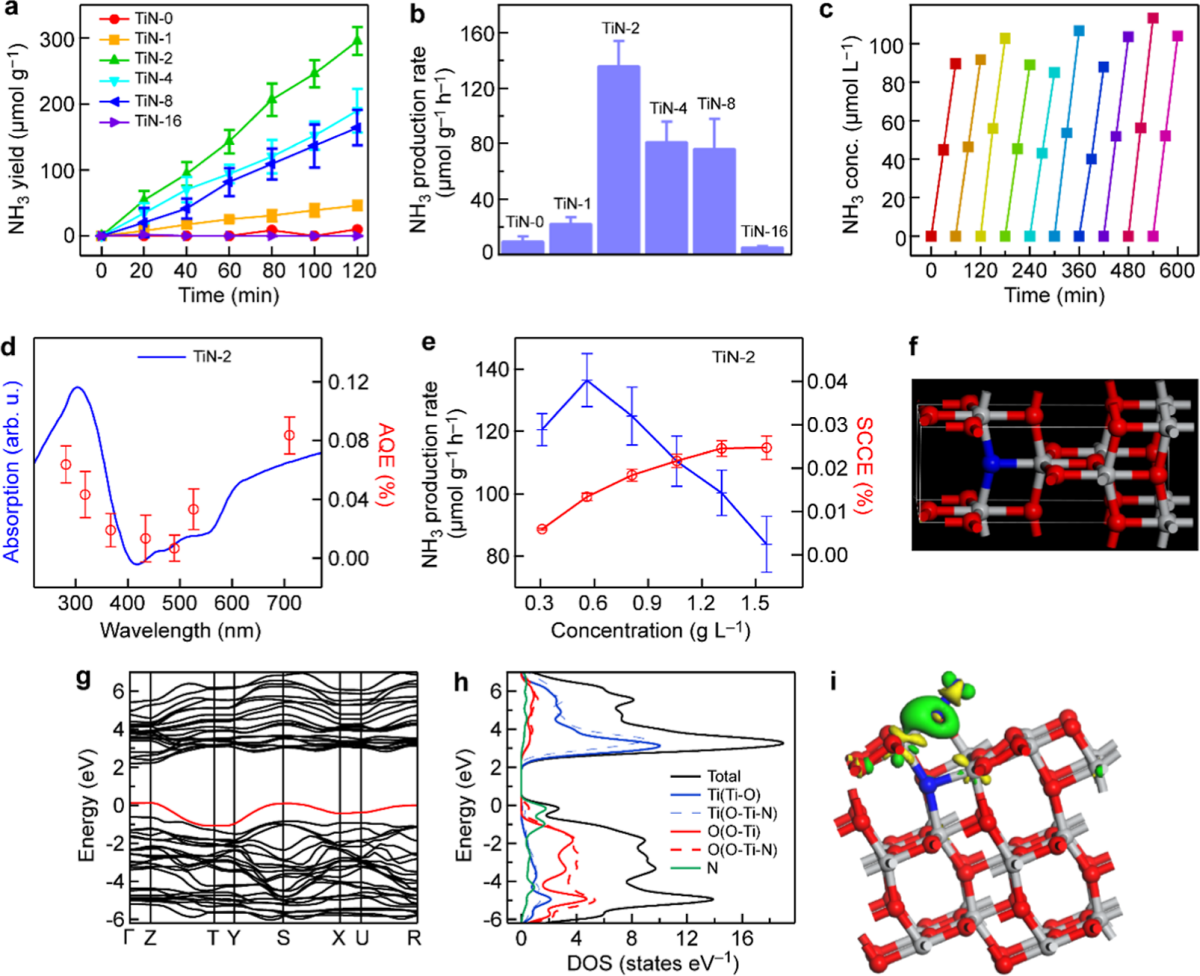
Photocatalytic nitrogen fixation with the TiN-*x* NPs. (a) Photocatalytic NH_3_ yields of the TiN-*x* NPs. (b) Photocatalytic nitrogen fixation rates of the TiN-*x* NPs. (c) Stability test of the TiN-2 NPs for photocatalytic nitrogen fixation. (d) AQEs of the TiN-2 NPs. (e) SCCEs of the TiN-2 NPs. (f) Structure model of TiN/TiO_2_. (g) DFT-calculated band structure of TiN/TiO_2_. (h) PDOS of TiN/TiO_2_. (i) Adsorption configuration and charge density difference of a N_2_ molecule adsorbed on TiN/TiO_2_ (adsorption energy: −0.86 eV; bond length: 1.151 Å).

The AQEs of the TiN-2 NPs were measured using monochromatic light filters of different wavelengths. They display a notable increase in the near-ultraviolet and visible regions, which is consistent with the trend of the light absorption spectrum of the TiN-2 NPs. The highest AQE reaches 0.08% under illumination with monochromatic light at the wavelength of 700 nm (Figure 4d). These AQEs can be attributed to the strong light absorption and efficient utilization of hot electrons by the TiN-2 NPs within the ultraviolet to the visible region. Apart from the AQE, the SCCE is also often used to evaluate the solar energy conversion capability of a photocatalyst. It serves as a more holistic metric for assessing the photocatalytic nitrogen fixation performance. The TiN-2 NPs give a noteworthy SCCE value of 0.02% (Figure 4e). The SCCE value reflects the overall solar energy conversion efficiency of a photocatalytic system, which improves with increasing photocatalyst loading amounts owing to enhanced light absorption. However, this improvement gets saturated at high photocatalytic loadings because of light scattering and saturated light absorption. In contrast, the mass-normalized NH_3_ production rate depends on the light absorption and reaction kinetics, which can be hindered by the limited light penetration and N_2_ diffusion at high photocatalyst loadings. As a result, with increasing photocatalyst loadings, the mass-normalized NH_3_ production rate decreases, while the SCCE is slightly improved through more efficient light utilization.

DFT calculations were carried out to investigate the electronic structure of TiN/TiO_2_ and the activation of N_2_ molecules. The anatase TiO_2_ without OVs exhibits a wide bandgap of 3.23 eV, which is consistent with the experiment (Figure S22a). The CB edge of the anatase TiO_2_ is mainly composed of the Ti 3d orbitals, while the VB edge is primarily originated from the O 2p orbitals (Figure S22b). The electronic structure of TiO_2_ with OVs were analyzed to elucidate the role of the OV for photocatalytic nitrogen fixation (Figure S23). OVs introduce defect energy levels, which result from the uncoordinated Ti 3d states at OVs. The defect energy levels are strongly spin-polarized, which enhances the activation of N_2_ molecules.^38^ Therefore, OVs can effectively catalyze the reduction of N_2_ through the strong N_2_ activation and the electron-capture effects. Moreover, the electronic states were examined using the structural model of a TiN/TiO_2_ hybrid (Figure 4f). Nitrogen doping gives energy states at the top of the VB, narrowing the bandgap from 3.23 eV for TiO_2_ to 2.08 eV (Figure 4g). The projected density of states (PDOS) analysis further confirmed that the VB edge of TiN/TiO_2_ is mainly composed of the N 2p orbitals (Figure 4h).

We further investigated the adsorption behavior of N_2_ molecules on the surface of the various TiO_2_ samples, including TiO_2_ without OVs, TiO_2_ with OVs, N-doped TiO_2_, and TiO_2_ with both OVs and N doping. When a N_2_ molecule is adsorbed, it might interact with Ti–N–O, Ti–O–Ti, Ti–N–OV, or Ti–OV–Ti sites. Figure S24 displays the calculated adsorption configurations and charge density differences for N_2_ adsorption on TiO_2_ without OVs, TiO_2_ with OVs, and N-doped TiO_2_, which correspond to Ti–O–Ti, Ti–OV–Ti, and Ti–N–O sites, respectively. For the TiN/TiO_2_ NP, the calculation was conducted for N_2_ adsorption at the Ti–N–OV site (Figure 4i). The calculation results reveal that the N_2_ chemisorption energies are significantly enhanced to −0.86 eV on the OVs of N-doped TiO_2_ and −0.89 eV on the OVs of TiO_2_. The bond length is also prominently elongated. The enhancement of N_2_ adsorption by OVs is consistent with the fact that the Ti atom at the OV site is strongly spin-polarized.^38^ The enhanced adsorption of N_2_ by OVs is also reflected by the charge density difference. When N_2_ molecules are adsorbed on OV sites, a substantial electron transfer occurs between the N_2_ molecule and the OV. As a result, the enhanced photocatalytic N_2_ fixation activity on TiN/TiO_2_ is originated from the superior light absorption induced by the plasmon resonance of TiN and the strong N_2_ activation capability facilitated by OVs on N-doped TiO_2_.

In addition to photocatalytic nitrogen fixation, we further investigated the photocatalytic hydrogen production of the TiN-*x* NPs. Among all the samples, the TiN-2 NPs also exhibit the highest hydrogen production performance (Figure 5a). The TiN-2 NPs gave a hydrogen production rate of 36.52 μmol g^–1^ h^–1^ when pure water was used as the solvent. Upon the use of methanol as a sacrificial agent, the hydrogen production rate of the TiN-2 NPs increased to 82.60 μmol g^–1^ h^–1^ (Figure 5b). Significantly, with the inclusion of 3 wt % Pt as a cocatalyst and 20 vt % of methanol as the hole sacrificial agent, the hydrogen production rate of the TiN-2 NPs reached an impressive rate of 2641.87 μmol g^–1^ h^–1^ (Figure 5b,c), which is 72.34 and 31.98 times higher than those of the previous two conditions, respectively. In our investigation of the oxidation products of the TiN-*x* NPs, the TiN-2 NPs exhibited the highest oxygen production rate of 127.58 μmol g^–1^ h^–1^, outperforming the other samples (Figure S25). Furthermore, the DFT calculations indicated that the formation of O_2_ from two O atoms represents the rate-determining step in the process (Figures 5d and S26). It is important to note that the oxygen generation shown in Figure S25 was observed during the photocatalytic nitrogen fixation tests, where no hole scavenger was used, and water oxidation to oxygen served as the counter reaction to notrogen reduction. In contrast, during the hydrogen evolution tests with methanol as a sacrificial agent, the oxidation of methanol consumed photogenerated holes, resulting in minimal oxygen generation. The selectivity of the photocatalytic reaction toward nitrogen fixation against hydrogen production is governed by the reaction conditions and the surface properties of the TiN-2 NPs. In the presence of N_2_ gas, the reduction of N_2_ to NH_3_ is favored owing to the enhanced adsorption and activation of N_2_ molecules on OV sites, as supported by the DFT calculations. Conversely, in an argon atmosphere with a hole scavenger like methanol, the reduction of water to hydrogen becomes the primary reaction.

**Figure 5:**
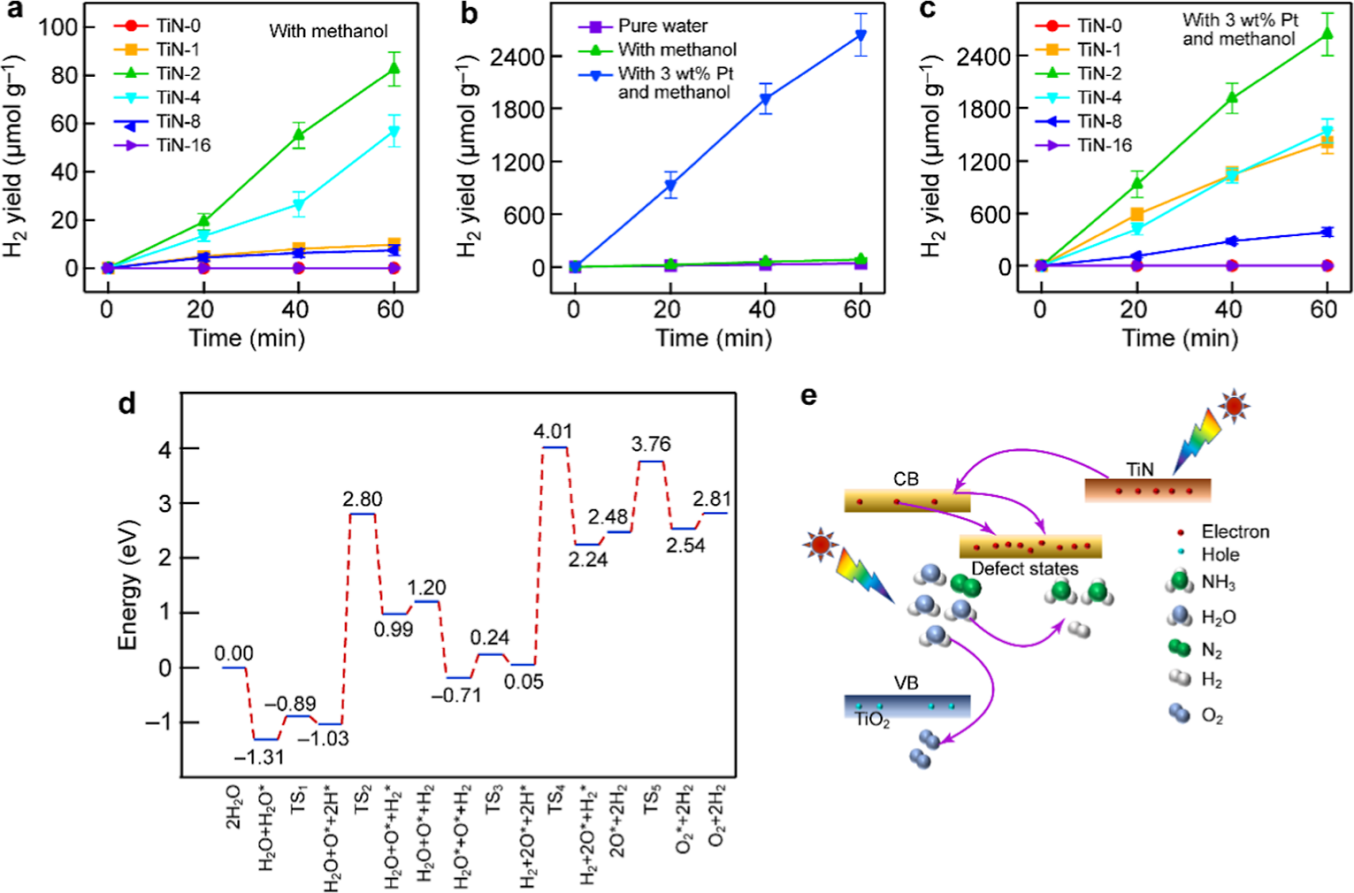
Photocatalytic hydrogen production of the TiN-*x* NPs and schematic of the photocatalytic mechanism over the TiN-2 NPs. (a) Photocatalytic hydrogen production of the TiN-*x* NPs with methanol as the sacrificial agent. (b) Comparison of the photocatalytic hydrogen production performance of the TiN-2 NPs under three different conditions. (c) Photocatalytic hydrogen production of the TiN-2 NPs with 3 wt % Pt as a cocatalyst. (d) Energy diagram for water splitting over the TiN/TiO_2_ NPs. (e) Schematic illustrating the photocatalytic mechanism over the TiN-2 NPs.

Based on the band structure of TiO_2_ and the work function of TiN, the mechanism of the plasmon-enhanced photocatalysis on the TiN-2 NPs is schematically shown in Figures 5e and S27. Unlike conventional metal–semiconductor hybrid plasmonic photocatalysts where only sufficiently energetic hot carriers can surpass the Schottky barrier at the interface, our newly constructed SBFPP has no Schottky barrier because the CB of TiO_2_ lies below the Fermi level of TiN (Figure 3c). The defect states present in the TiN-2 NPs are also located below the Fermi level of TiN because the defect states of TiO_2_ are usually positioned below its CB.^39–41^ Electrons located in the VB of TiO_2_ are excited to the CB under light illumination while hot electrons are also generated within the plasmonic TiN component. These photoexcited electrons from both pathways subsequently transfer to the defect states associated with the activation sites to participate in the photocatalytic nitrogen fixation or hydrogen generation reaction.

The utilization of solar-driven nitrogen fixation through photocatalysis encounters significant challenges, such as the unavoidable water light absorption in the NIR region and the slow mass transfer kinetics of the reactants. These obstacles severely hinder the practical implementation and widespread applications of photocatalytic nitrogen fixation. To address these issues, we further developed a floatable biphasic plasmonic photocatalytic membrane system, which consisted of a porous poly­(vinyl alcohol) (PVA) film and the TiN-2 photocatalyst. The PVA porous film was fabricated by freeze-drying, with the TiN/TiO_2_ photocatalyst nanoparticles embedded in the pores (Figure 6a,b). The TiN/TiO_2_ photocatalyst nanoparticles are adhered to the surface of the PVA network (Figure 6c). The pure PVA film displays a white appearance (Figure 6d), while the PVA film with the encapsulated photocatalyst appears grayish hue (Figure 6e). The absorption spectra of the PVA films with varied amounts of the loaded photocatalyst are presented in Figure 6f. The water layer thickness on the film surface was adjusted by controlling the height at which the porous film was floated on the water, giving rise to a very thin water layer on the film surface. This thin water layer minimizes water-induced light attenuation, effectively enhancing the efficiency of the photocatalytic nitrogen fixation process. The experimental results of the TiN-2 (40 mg)/PVA film demonstrate a high NH_3_ yield of 606.3 μmol g^–1^ h^–1^, where air was used as the feed gas (Figure 6g). The NH_3_ yield is superior to those of most photocatalytic nitrogen fixation catalysts (Table S3). The TiN-2 (40 mg)/PVA film improves the photocatalytic nitrogen fixation performance by 4.4 times compared to the TiN-2 NPs. Our investigation into the structure and dynamics of water molecules within the TiN-2/PVA film unveils the presence of three distinct types of water molecules: bound water, intermediate water, and free water (Figure 6h,i). These water molecules interact with the PVA network through different types of hydrogen bonding, significantly affecting the solar absorption and catalytic reaction. The structural configuration of water molecules in the thin water layer is remarkably distinct from that of bulk water, further enhancing the mass transfer kinetics of N_2_ molecules and promoting the photocatalytic activity. Consequently, the floatable biphasic solar-driven nitrogen fixation film markedly improves the efficiency and practical applicability of photocatalytic nitrogen fixation. The superior photocatalytic activity and efficient solar energy utilization presented by this film system open new prospects and possibilities in the field of photocatalytic nitrogen fixation.

**Figure 6:**
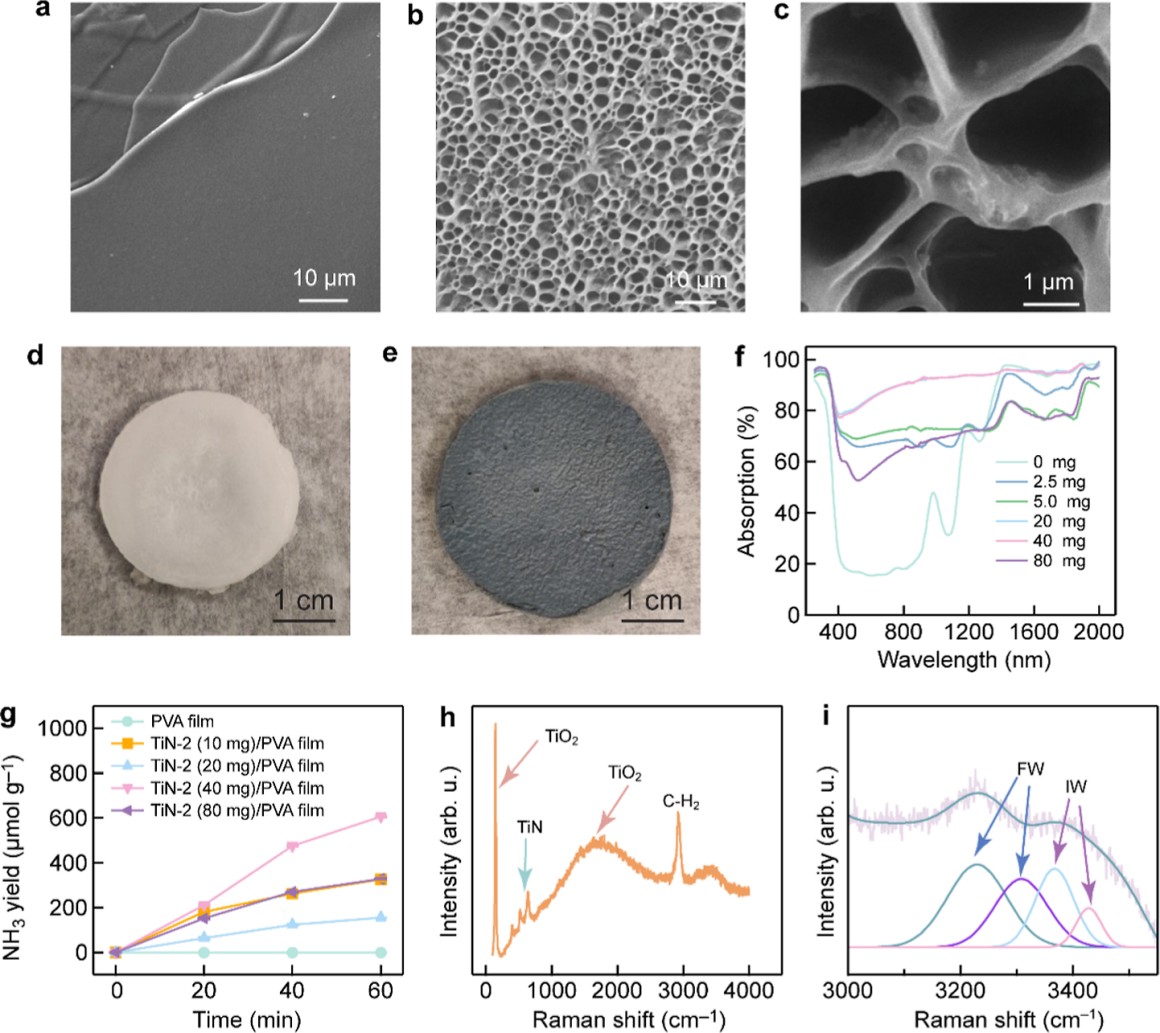
Photocatalytic performance and characterization of the TiN-2/PVA films. (a) SEM image of the prefreeze-dried PVA film. (b) SEM image of the post-freeze-dried PVA film. (c) SEM image of the TiN-2/PVA film. (d) Photograph of the pristine PVA film. (e) Photograph of the TiN-2/PVA film. (f) Diffuse reflectance spectra of the TiN-2/PVA films with varied amounts of the loaded photocatalyst. (g) Photocatalytic nitrogen fixation performances of the biphasic films loaded with different amounts of the TiN-2 NPs. (h) Raman spectra of the biphasic film. (i) Raman spectra of the pure PVA film.

## 3 Conclusion

In conclusion, we have produced the TiN/TiO_2_ NPs through a simple oxidation process from commercially available TiN nanoparticles and demonstrated that the TiN/TiO_2_ NPs constitute a new type of SBFPP. The TiN/TiO_2_ NPs exhibit an excellent photocatalytic nitrogen fixation performance. They possess a substantial number of doping defects and OVs, which result in the creation of defect states below the CB. Notably, the low work function of the TiN component allows the defect states to be located below the Fermi level of TiN, enabling hot electrons generated in TiN to efficiently migrate to these defect states and actively participate in the photocatalytic reaction. This efficient transfer mechanism significantly enhances the utilization efficiency of hot electrons during the photocatalytic process, which has been proved by optical measurements, photocatalytic tests, and DTF calculations. Furthermore, the TiN-2 NPs demonstrate versatile functionality. They are not only effective for photocatalytic nitrogen fixation but also for efficient hydrogen production when combined with Pt as the cocatalyst. In addition, the photocatalytic film formed by integrating TiN/TiO_2_ with a porous PVA film addresses the issue of strong absorption of NIR light by water during the photocatalytic process, thereby improving the photocatalytic nitrogen fixation performance. This study offers a promising avenue for the rational design of photocatalytic platforms and thus for future practical applications in the field of photocatalysis.

## 4 Methods

### 4.1 Chemicals

The TiN NPs were purchased from Aladdin (CAS no. 25583-20-4). Absolute ethanol (99.97 wt %) was acquired from VWR Chemicals. Acetone was purchased from Fisher Scientific. Chloroplatinic acid was purchased from Aladdin (CAS no. 26023-84-7).

### 4.2 Characterization

SEM imaging was performed on a JEOL JSM7800F microscope. The operation pressure and acceleration voltage were 9.6 × 10^–5^ Torr and 20 kV, respectively. The elemental compositions of the samples were determined with an Oxford EDX analysis system on the same microscope. TEM imaging was conducted on an FEI Tecnai Spirit 12 microscope under an acceleration voltage of 120 kV. XPS was carried out on a PerkinElmer PHI 5000C system to analyze the elemental compositions and valence states of the samples. XRD patterns were acquired on a Rigaku SmartLab diffractometer equipped with Cu Kα radiation. TGA was carried out on a PerkinElmer TGA 6 thermogravimetric analyzer to measure the weight changes of the samples at a constant heating rate. The diffuse reflectance absorption spectra of the samples were measured on a PerkinElmer Lambda 950 ultraviolet/visible/NIR spectrophotometer in the integrating sphere mode. EPR spectra were recorded on a JES-FA300 electron spin resonance spectrometer.

### 4.3 Electrochemical Measurements

The electrochemical measurements were performed with a three-electrode system on an electrochemical workstation (CHI 760E, Shanghai Chenhua). The photocatalyst (5 mg) in Nafion solution (0.05 mL) and deionized water (0.95 mL) was deposited on a transparent fluorine-doped tin oxide (FTO) glass slide (1 × 1 cm^2^) to function as the working electrode. A Pt plate and the standard Ag/AgCl electrode were employed as the counter and reference electrodes, respectively. Aqueous Na_2_SO_4_ (0.1 M, 80 mL) was employed as the electrolyte.

Mott–Schottky plots were derived using the impedance-potential technique. The relationship between the interfacial capacitance (*C*) connecting the working electrode and the electrolyte and the imaginary component of the impedance (*Z*_*i*_) can be described by the equation below

1 $$C=-\frac{1}{{2\pi fZ}_{i}}$$

The frequency employed for the impedance-potential measurement was set at *f* = 1000 Hz. The determination of the flatband potential (*E*_fb_) and apparent carrier concentration (*n*) was accomplished using the following relationship

2 $$\frac{1}{C^{2}}=\frac{2}{eA^{2}\varepsilon_{0}\varepsilon_{r}n}\times \left(E-E_{fb}-\frac{k_{B}T}{e}\right)$$

where *A* represents the interfacial area between the working electrode and the electrolyte, *e* denotes the elementary electron charge, ε_0_ corresponds to the vacuum permittivity, ε_r_ denotes the relative permittivity of the electrode material at the frequency *f*, *n* represents the carrier concentration, *E* denotes the applied voltage, *k*_B_ stands for the Boltzmann constant, and *T* is the temperature in Kelvin. A linear relationship was observed by plotting *A* ^2^/*C* ^2^ against *E*. The flatband potential (*E*_fb_) was obtained from the intercept on the *E* axis, whereas the electron carrier concentration (*n*) was deduced according to the slope of the linear relationship.

EIS was acquired using the AC impedance technique. The initial voltage *E* was set at a value that was 0.5 V higher than the open-circuit potential. The frequency was set in the range of 0.01–100,000 Hz.

The photocurrent curves were measured with a 300 W xenon lamp (PLS-SXE300C, Beijing Perfectlight Technology) equipped with an AM 1.5 sunlight filter. A continuous flow of either argon or N_2_ was introduced into the electrolyte solution. The light was periodically turned off every 20 s with the initial voltage *E* set at 0.5 V. The role of nitrogen in the photocatalytic reaction process was assessed through the analysis of the difference in the photocurrent profiles obtained under the argon and nitrogen atmospheres.

### 4.4 Photocatalytic Nitrogen Fixation Tests

Photocatalytic nitrogen fixation reactions and associated assessments were conducted following our reported procedure.^34^ The photocatalytic sample (40 mg) was first weighed and subsequently placed into a centrifuge tube containing deionized water (50 mL). The sample was then subjected to centrifugation and washing before being introduced into a custom-made photocatalytic nitrogen fixation reactor. Deionized water (80 mL) was subsequently added to the reactor and uniformly mixed by ultrasonication. No hole scavenger was used during the photocatalytic nitrogen fixation tests. The concurrent reaction was the oxidation of water to give oxygen. The photocatalytic nitrogen fixation reactor was then placed on a magnetic stirrer to maintain a constant stirring speed, while high-purity N_2_ was continuously introduced into the container at a steady flow rate of 50 mL min^–1^. The light intensity of the xenon lamp was adjusted to 1000 W m^–2^ using an optical power meter to simulate sunlight. The xenon lamp was equipped with various filters to investigate the effect of monochromatic light at different wavelengths on the photocatalytic nitrogen fixation reaction. To monitor the production of ammonia, the reaction solution (1.5 mL) was collected every 10 min using a syringe, with the collection before light illumination considered as the starting point of the photocatalytic reaction. The collected reaction solution was subjected to centrifugation. The obtained supernatant was analyzed to determine the concentration of the produced ammonia.

### 4.5 Ammonia Detection

The concentration of ammonia produced in the photocatalytic reaction was determined by the chromogenic method with Nessler’s reagent.^11^ Nessler’s reagent is composed of K_2_HgI_4_ (0.09 mol L^–1^), KOH (2.5 mol L^–1^), and deionized water. It reacts with ammonia according to the reaction

3 $$NH_{3}+2{\left[HgI_{4}\right]}^{2-}+3OH^{-}\to HgOHg\left(NH_{2}\right)I+7I^{-}+2H_{2}O$$

The color of the solution turns from pale to deep yellow with the increase of the NH_3_ concentration. HgOHg­(NH_2_)I is a yellow transparent solution at low concentrations. In a typical process, the analyte solution (0.5 mL) was first mixed with KNaC_4_H_4_O_6_ solution (0.2 mol L^–1^, 0.25 mL), which would minimize the interference of other ions, such as Fe^3+^, Co^2+^, Ni^2+^, Cr^3+^, Ag^+^, and S^2–^, in the subsequent reaction. Nessler’s reagent (0.25 mL) was then added to the mixture solution, and the entire resultant mixture solution was kept for 15 min to ensure the complete reaction. The ammonia concentration was calculated by monitoring the absorbance at 425 nm according to the pre-established linear relationship between the ammonia concentration and the absorbance recorded on an ultraviolet/visible/near-infrared spectrophotometer.

### 4.6 Photocatalytic Hydrogen Production Tests

The photocatalytic hydrogen and oxygen production reactions and associated assessments were conducted following our reported procedure.^11^ The experimental conditions were similar to those of photocatalytic nitrogen fixation, except that when methanol was used as a sacrificial agent, the solution was changed from deionized water (50 mL) to an aqueous methanol solution with a methanol content of 20 vol %. The H_2_ and O_2_ amounts were directly measured on a gas chromatograph (CEAULIGHT GC-7900, TCD detector, Ar carrier gas).

### 4.7 ^15^N_2_ Isotope Labeling Experiments

The ^15^N_2_ isotope labeling experiments were conducted with nuclear magnetic resonance (NMR, Varian Inova, 500 MHz, USA). First, the reaction solution (5 mL) was filtered with a 0.22 μm membrane to remove the photocatalyst nanoparticles. The obtained solution was then adjusted with a H_2_SO_4_ solution (6 mol L^–1^) to pH = 1.2. DMSO-*d*_6_ (100 μL) was subsequently added to the acidified solution (1 mL), which was followed with thorough mixing.

### 4.8 DFT Calculations

The DFT calculations were performed using the Vienna Ab initio Simulation Package (VASP) software.^42^ The generalized gradient approximation by Perdew, Burke, and Ernzerhor^43^ was employed for the exchange–correlation energy. The ionic cores were described by projector-augmented wave potentials.^44^ For the catalytic reaction calculation, the (101) facet was used to model the surface of the TiO_2_ NPs because the (101) facets of anatase TiO_2_ have the lowest energy and thus are the most exposed surfaces.^45^ The (101) facet was constructed using a (2 × 2) supercell, with 18 atomic layers. For TiO_2_ with OVs, an OV was created on the surface of the (2 × 2) supercell. For N-doped TiO_2_, a surface oxygen atom was replaced by a nitrogen atom. The N-doped TiO_2_ with OVs was constructed by replacing an oxygen atom with a nitrogen atom and removing an adjacent oxygen atom. The vertical separation between the successive slabs was set at 1.5 nm. All structural optimizations were performed using a cutoff at 400 eV for the plane-wave basis set. The Brillouin zones were sampled with (3 × 5 × 1) Monkhorst–Pack grids.^46^ For the calculation of the electronic states, the original cell of anatase TiO_2_ was used. The N-doped TiO_2_ and TiO_2_ with OVs were modeled by replacing an oxygen atom with a nitrogen atom and removing an oxygen atom, respectively. The energy bands and density of states were calculated with DFT + *U*.

### 4.9 Preparation of the TiN/TiO_2_ NPs

The TiN/TiO_2_ NPs were synthesized by a simple thermal treatment method. The commercially available TiN NPs were heated in a tube furnace at an oxidation temperature of 400 °C and holding durations of 0–16 h. The obtained samples are denoted as TiN-*x*, where *x* represents the holding time.

### 4.10 Preparation of the TiN/TiO_2_/PVA Films

The preparation of the TiN/TiO_2_/PVA films commenced with the dissolution of PVA (375 mg) and polyethylene glycol (PEG, 30 mg) in deionized water (5 mL). Glutaraldehyde (46.88 μL) was subsequently added to the above solution, followed by uniform mixing by ultrasonication. This mixture was labeled as Solution A. Separately, the TiN-2 sample (40 mg) was dispersed in deionized water (295.6 μL), followed by ultrasonication to achieve homogeneity. This TiN-2 solution was then combined with HNO_3_ (10 wt %, 204.4 μL), resulting in Solution B. Solutions A and B were then thoroughly mixed in a 50 mL beaker. The mixed solution was further subjected to ultrasonication, followed by solidification overnight in a freezer compartment. The solidified mixture was finally freeze-dried for 24 h to obtain the TiN/PVA film.

## Supplementary Material



## Notes

Thermogravimetric curve; photographs of the TiN NPs; SEM and TEM images of the TiN-*x* NPs; BET test; O 1s XPS spectra; elemental compositions; zeta potential tests; absorption spectra; Tauc plots; Mott–Schottky plot; photocurrent responses; EPR spectra; photographs of the photocatalytic device; linear relationship for the determination of the ammonium concentration; time-dependent production of ammonia; ^1^H NMR spectra; verification of the stability of the TiN-2 NPs; XPS spectra of Ti 2p and O 1s before and after the photocatalytic reaction with the TiN-2 NPs; DFT-calculated band structure and DOS; adsorption configurations and charge density differences; oxygen production rates; adsorption configurations and transition states; band energy diagram; band structures; photocurrent responses; NH_3_ yields reported in representative photocatalytic N_2_ fixation works (PDF)
