# Supplementary material for: Dual-Functional Schottky-Barrier-Free Plasmonic TiN/TiO2 Photocatalyst for Efficient NH3 and H2 Production
Source: ACS Appl Mater Interfaces. 2025 Jun 13;17(25):36773–83. doi: 10.1021/acsami.5c06863 (PMC12203463; doi:10.1021/acsami.5c06863)
Supplement: Supplementary file 1 [file am5c06863_si_001.pdf]

Supporting Information

Dual-functional Schottky-barrier-free Plasmonic TiN/TiO_2 Photocatalyst for Efficient NH_3 and H_2 Production

Xiaopeng Bai,[1,†] Ke An,[1,†] Lingyu Jia,[1] Yanzhen Guo,[4] Jingtian Hu,[1] Guangri Jia,[3] Ruibin Jiang,[2,] Jianfang Wang,[1,*] and Jimmy C. Yu[3]*

[1]Department of Physics, The Chinese University of Hong Kong, Shatin, Hong Kong SAR 999077, China

[2]Shaanxi Key Laboratory for Advanced Energy Devices, Shaanxi Engineering Lab for Advanced Energy Technology, School of Materials Science and Engineering, Shaanxi Normal University, Xi'an, Shaanxi 710119, China

[3]Department of Chemistry, The Chinese University of Hong Kong, Shatin, Hong Kong SAR 999077, China

[4]Henan Provincial Key Laboratory of Nanocomposites and Applications, Institute of Nanostructured Functional Materials, Huanghe Science and Technology College, Zhengzhou, Henan 450006, China

†X.P.B. and K.A. contributed equally to this work.

*Email: rbjiang@snnu.edu.cn; jfwang@phy.cuhk.edu.hk

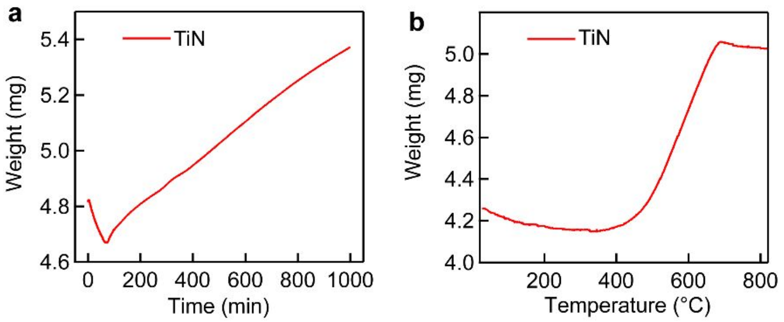

Figure S1. Thermogravimetric analysis of the TiN NPs. (a) Variation of the weight of the TiN NPs as a function of time. (b) Variation of the weight of the TiN NPs as a function of temperature. The heating rate was 5 °C min^{-1}. The temperature range was 30 to 800 °C.

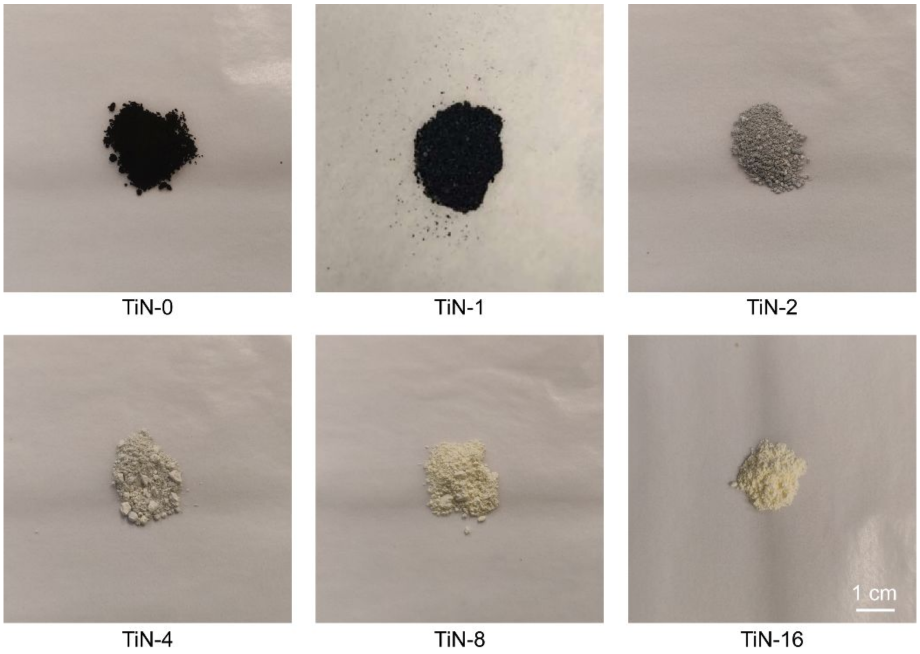

Figure S2. Photographs of the TiN NPs treated at 400 °C for different holding durations.

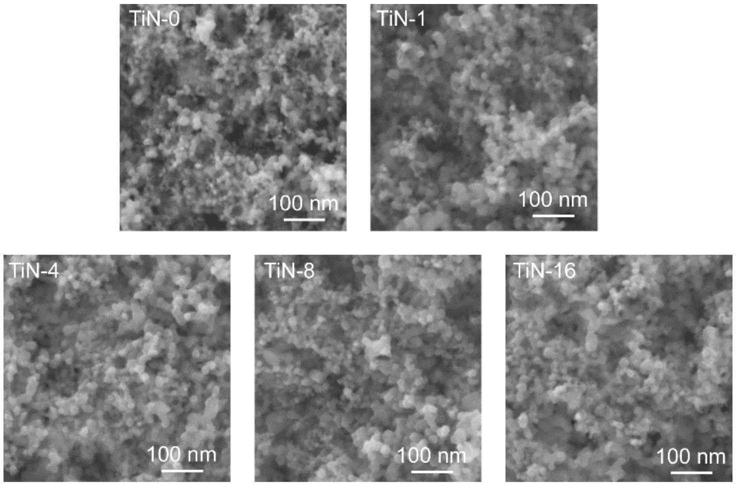

Figure S3. SEM images of the TiN-*x* NPs treated for different holding durations.

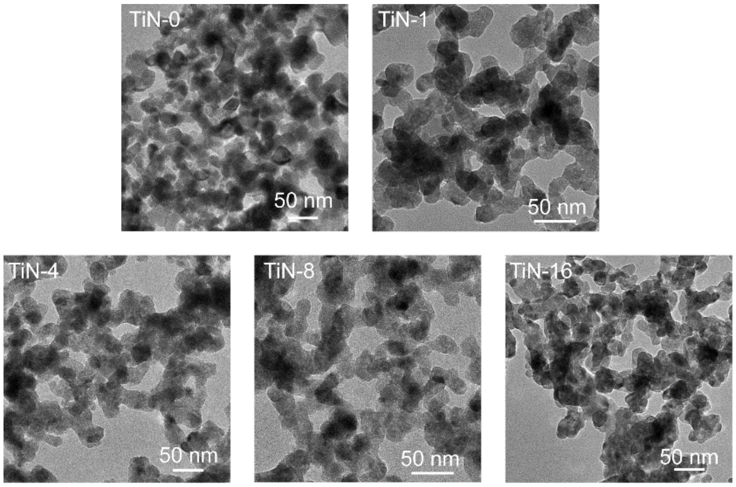

Figure S4. TEM images of the TiN-*x* NPs treated for different holding durations.

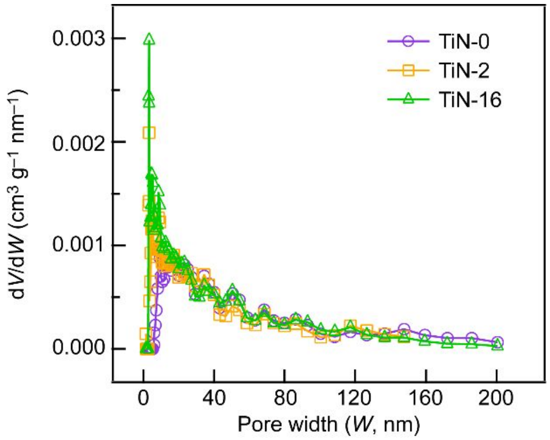

Figure S5. BET surface areas of the TiN-0, TiN-2, TiN-16 NPs.

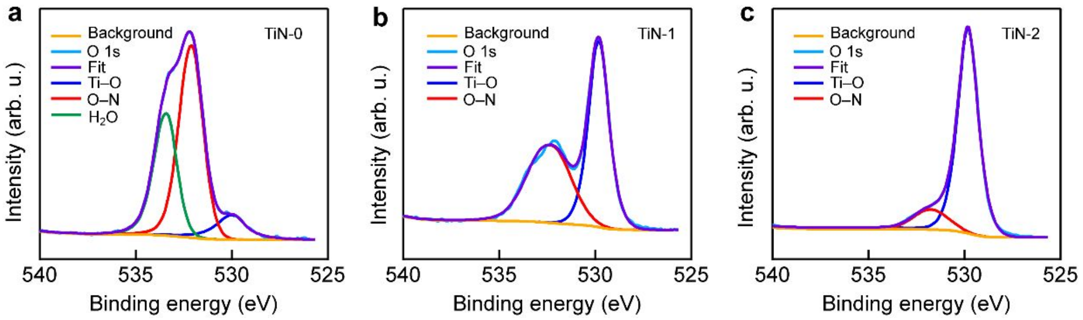

Figure S6. O 1s XPS spectra of the TiN-x NPs. (a) O 1s XPS spectra of the TiN-0 NPs. (b) O 1s XPS spectra of the TiN-1 NPs. (c) O 1s XPS spectra of the TiN-2 NPs.

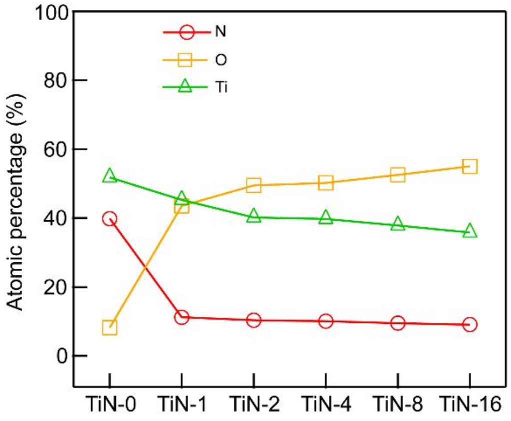

Figure S7. Elemental compositions of the TiN-x NPs determined by EDX.

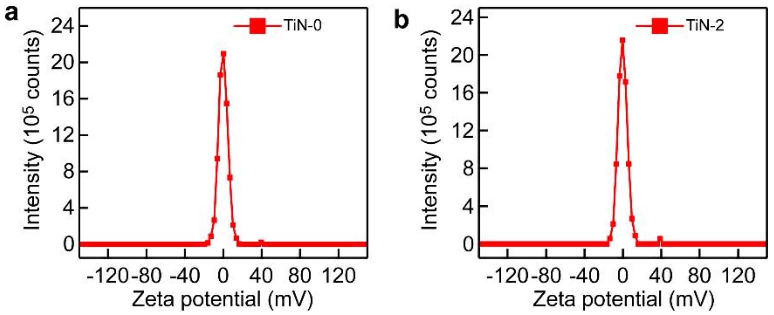

Figure S8. Zeta potential tests. (a) TiN-0 NPs. (b) TiN-2 NPs.

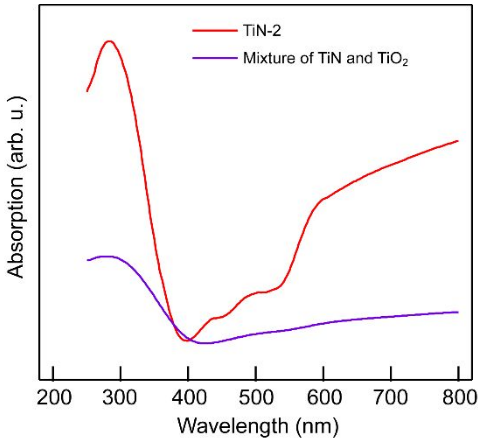

Figure S9. Absorption spectra of the TiN-2 NPs and physical mixture of the TiN and TiO_2 NPs.

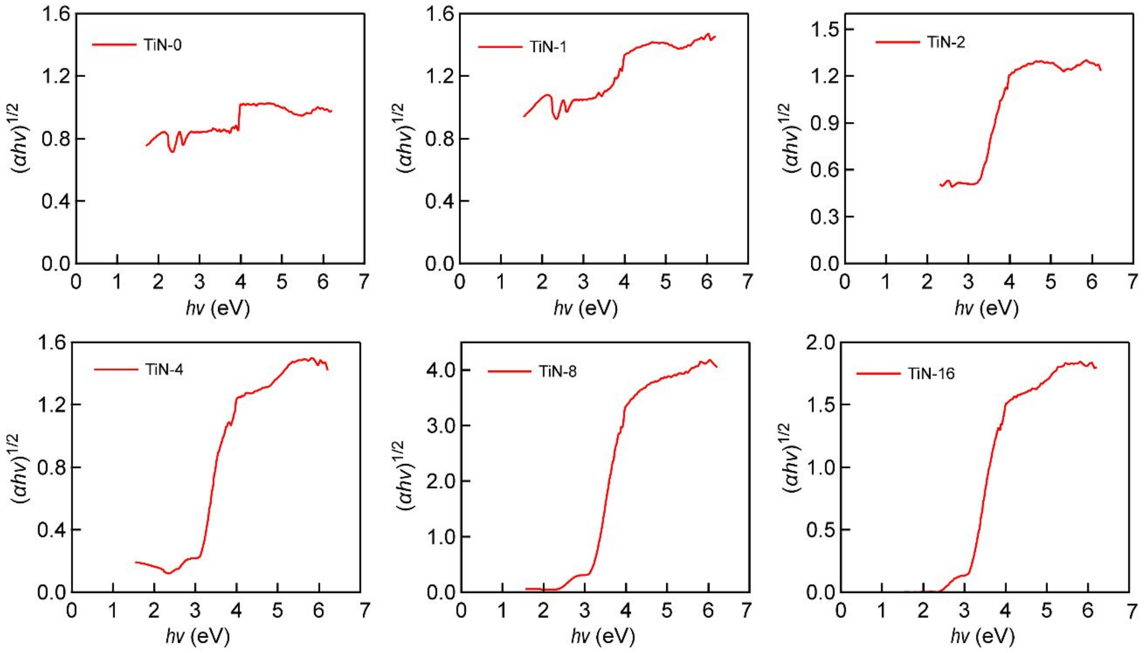

Figure S10. Tauc plots of the TiN-*x* NPs.

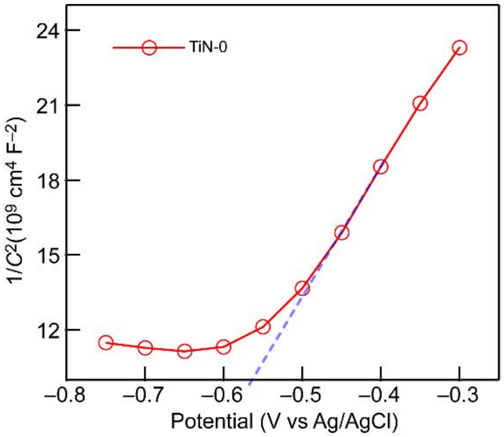

Figure S11. Mott–Schottky plot of the TiN-0 NPs.

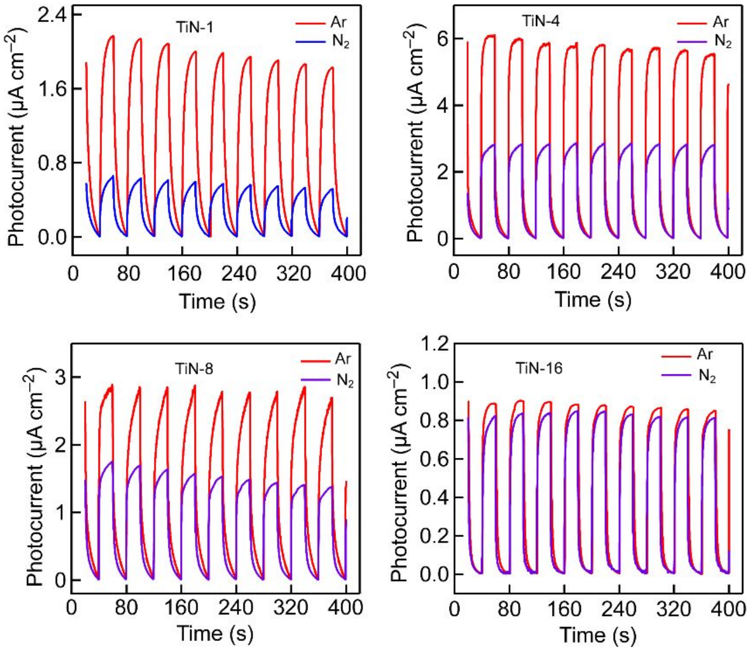

Figure S12. Photocurrent responses of the TiN-1 NPs, TiN-4 NPs, TiN-8 NPs, and TiN-16 NPs.

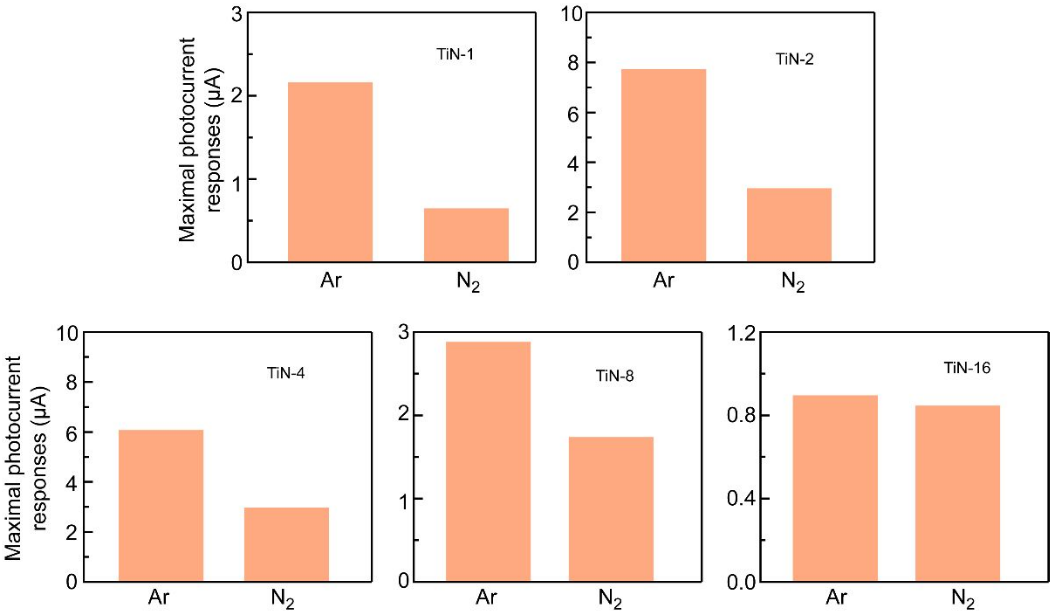

Figure S13. Photocurrent responses of the TiN-*x* NPs.

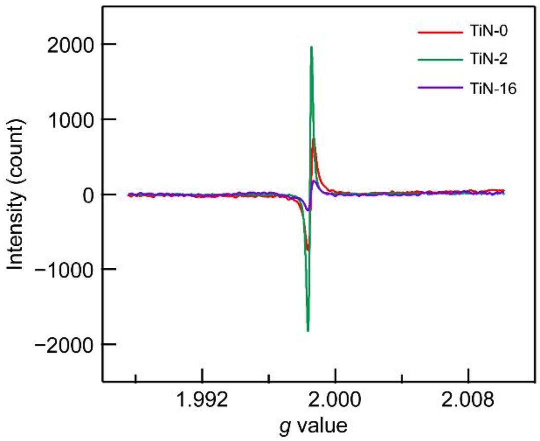

Figure S14. EPR spectra of the TiN-0 NPs, the TiN-2 NPs, and the TiN-16 NPs.

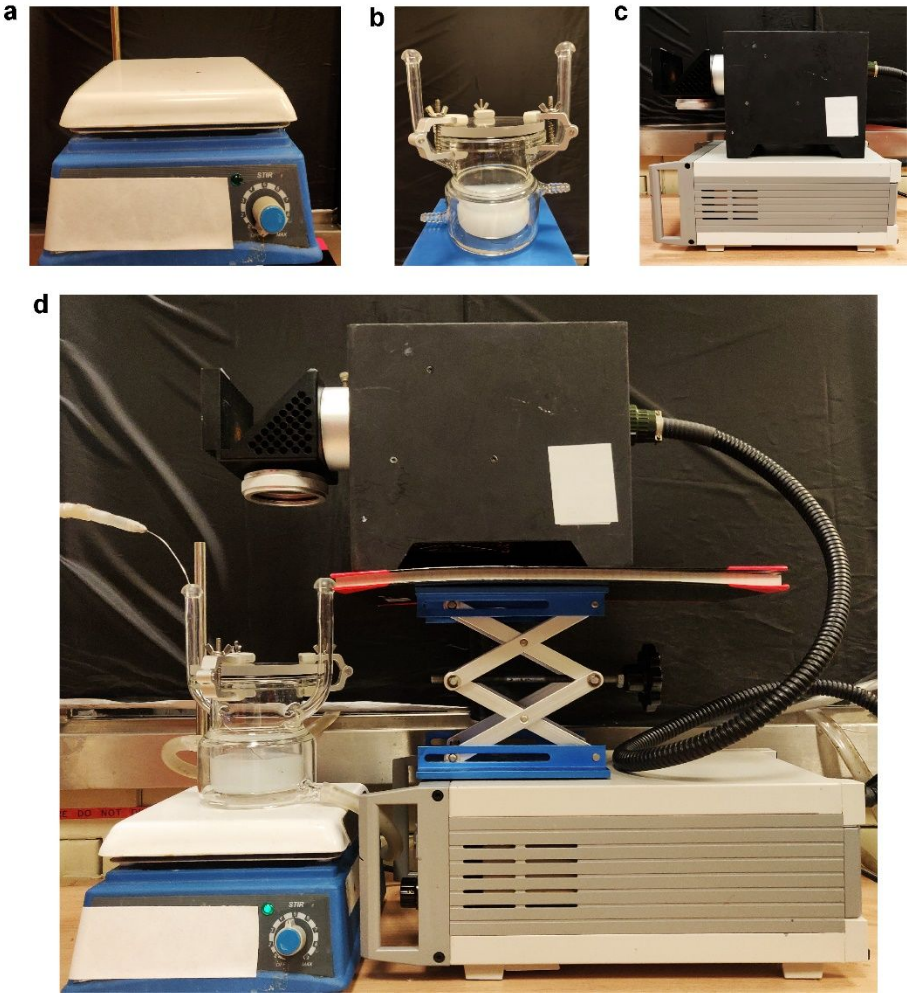

Figure S15. Photographs of the photocatalytic device. (a) Tachometer. (b) Photocatalytic reactor. (c) Xenon lamp. (d) Photocatalytic setup.

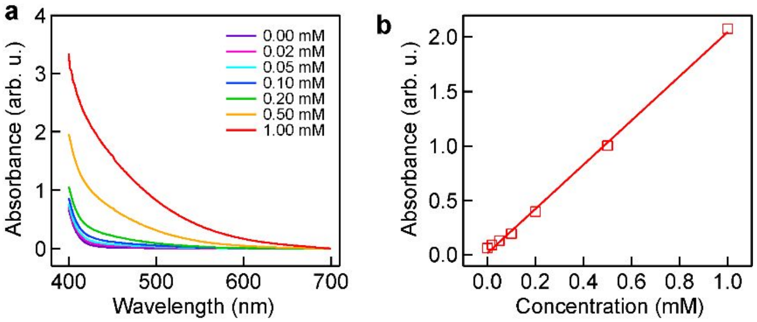

Figure S16. Linear relationship for the determination of the ammonium concentration. (a) Absorption spectra of the standard test solutions with different ammonium ion concentrations. (b) Absorbance value at 425 nm as a function of the ammonium ion concentration.

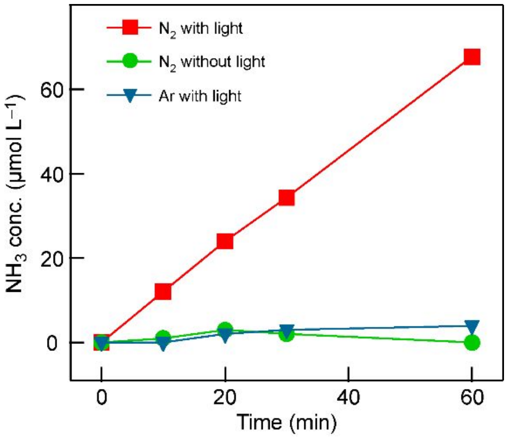

Figure S17. Time-dependent production of ammonia for the TiN-2 NPs under different conditions.

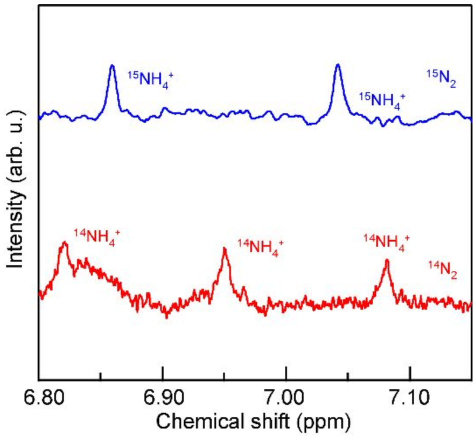

Figure S18. ^{1}H NMR spectra of the photocatalytic nitrogen fixation solutions. The reactions lasted for 2 h with the TiN-2 NPs in $^{14}N_2$ and $^{15}N_2$ atmospheres.

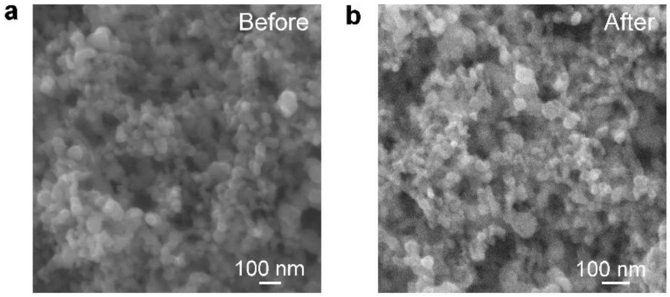

Figure S19. SEM images of the TiN-2 NPs before and after the photocatalytic nitrogen fixation reaction. (a) Before the photocatalytic nitrogen fixation reaction. (b) After the photocatalytic nitrogen fixation reaction.

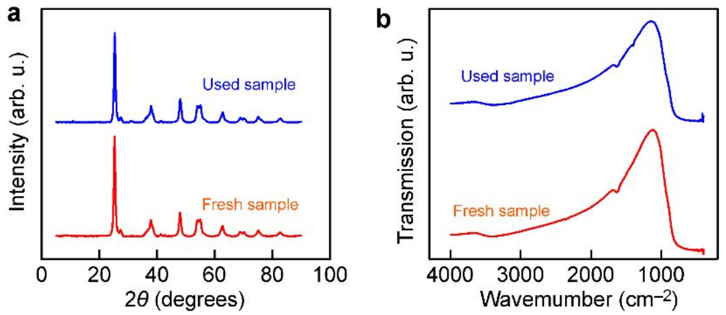

Figure S20. Verification of the stability of the TiN-2 NPs. (a) XRD patterns before and after the photocatalytic nitrogen fixation reaction. (b) Infrared spectra before and after the reaction.

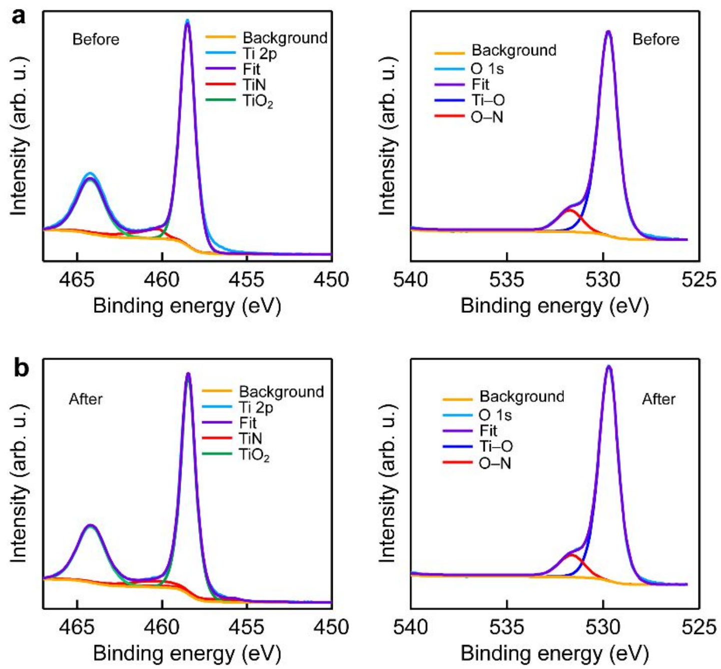

Figure S21. XPS spectra of Ti 2p and O 1s before and after the photocatalytic reaction with the TiN-2 NPs. (a) Before the photocatalytic reaction. (b) After the photocatalytic reaction.

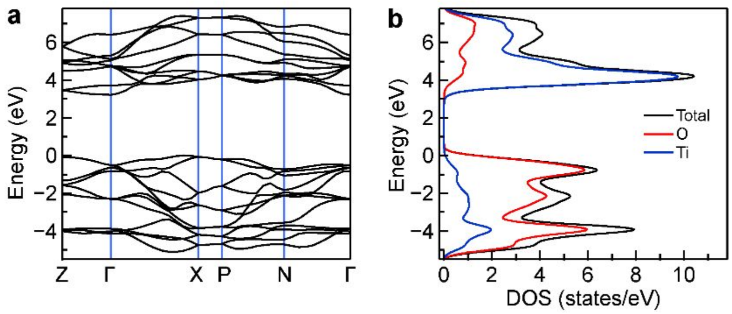

Figure S22. DFT-calculated band structure and density of states (DOS) of the anatase TiO_2. (a) DFT-calculated band structure of anatase TiO_2. (b) DOS of anatase TiO_2.

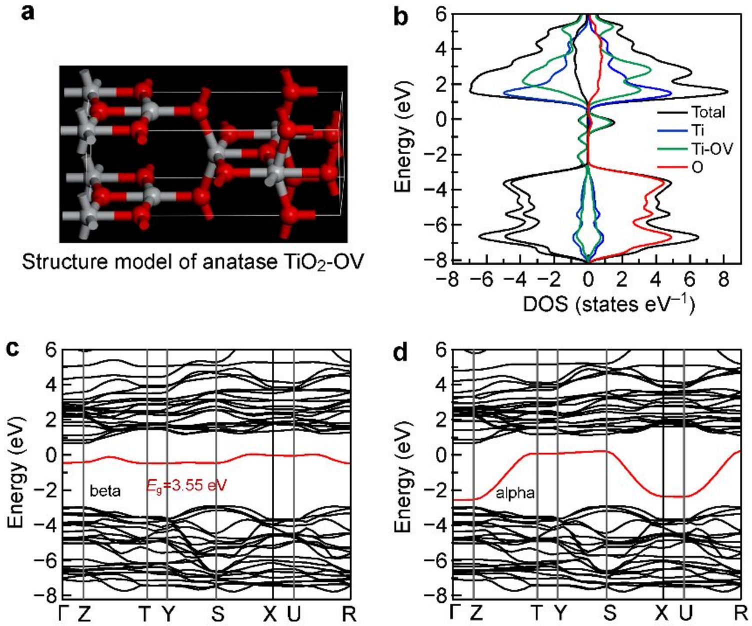

Figure S23. DFT calculation for anatase TiO_2-OVs. (a) Structural model. (b) Total and projected DOSs. (c,d) DFT-calculated band structure. Alpha and beta indicate the spin-up and down directions of electrons, respectively.

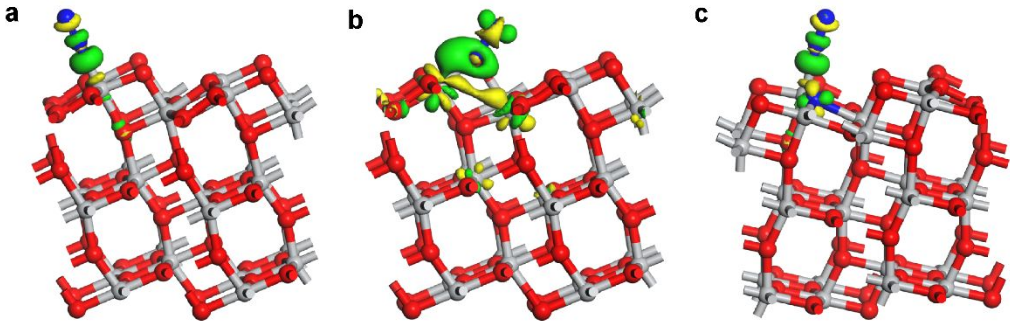

Figure S24. Adsorption configurations and charge density differences of a N_2 molecule adsorbed on the different structure models. (a) TiO_2 without OVs (adsorption energy: –0.49 eV; bond length: 1.137Å). (b) TiO_2 with OVs (adsorption energy: –0.89 eV; bond length: 1.153Å). (c) N-doped TiO_2 (adsorption energy: –0.44 eV; bond length: 1.136Å).

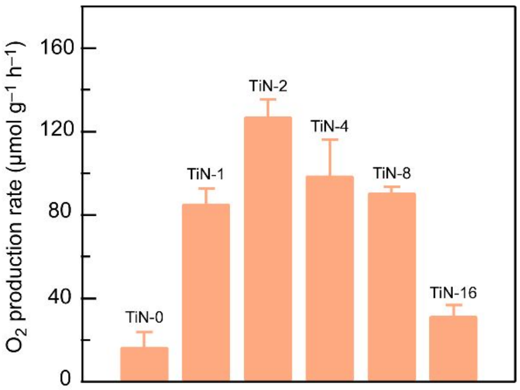

Figure S25. Oxygen production rates of the TiN-*x* NPs.

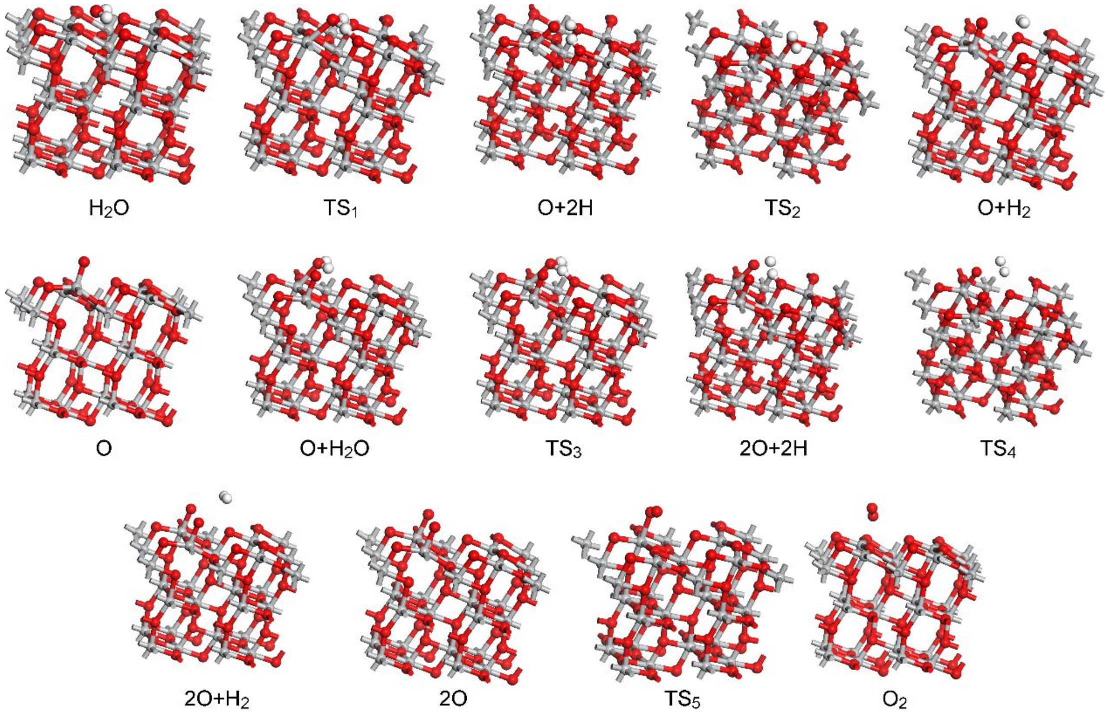

Figure S26. Adsorption configurations and transition states (TSs) for water splitting on the TiN-*x* NPs. TS_1 to TS_5 represent the critical TSs in the reaction pathway. TS_1 corresponds to the transition from the H_2O molecule to the O + 2H configuration, TS_2 from O + 2H to O + H_2, TS_3 from O + H_2O to 2O + 2H, TS_4 from 2O + 2H to 2O + H_2, and TS_5 leads to the formation of molecular oxygen (O_2) from the 2O intermediate.

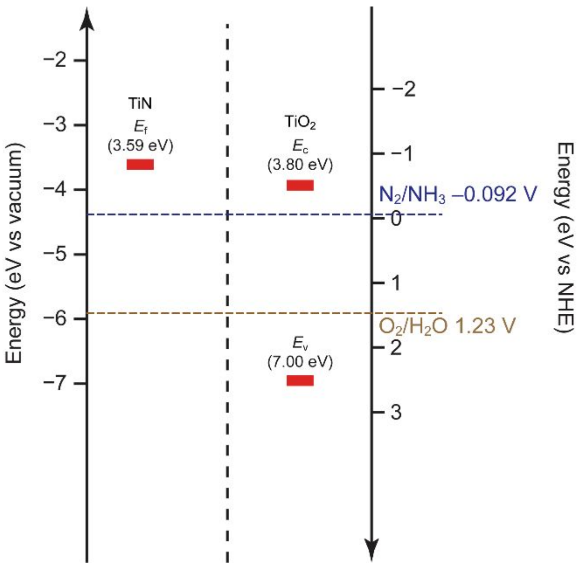

Figure S27. Band energy diagram of the TiN-2 NPs.

Table S1. Band Structures of the TiN-*x* NPs

Sample	Bandgap (eV)	E_c (eV, vs NHE)	E_v (eV, vs NHE)
TiN-0	/	/	/
TiN-1	2.65	−0.58	2.07
TiN-2	2.80	−0.57	2.23
TiN-4	3.05	−0.53	2.52
TiN-8	3.10	−0.6	2.50
TiN-16	3.20	−0.7	2.50

Table S2. Photocurrent Responses of the TiN-*x* NPs

Photocatalyst	Maximal photocurrent response in Ar (μA)	Maximal photocurrent response in N_2 (μA)	Reduced percentage (%)
TiN-1	2.17	0.66	69.7
TiN-2	7.76	3.01	61.2
TiN-4	6.12	2.87	53.1
TiN-8	2.89	1.75	39.5
TiN-16	0.90	0.85	5.6

Table S3. NH_3 Yields Reported in Representative Photocatalytic N_2 Fixation Works

Photocatalyst	Light source	Reaction conditions	NH_3 yield (μmol g_{cat}^{-1} h^{-1})	Reference
TiN-2	300 W xenon lamp, AM 1.5	40 mg catalyst, 80 mL H_2O, non-isothermal, N_2	136	This work
TiN-2/PVA	300 W xenon lamp, AM 1.5	40 mg catalyst, 80 mL H_2O, non-isothermal, N_2	606	This work
Au/TiO_2-OV	300 W xenon lamp, $\lambda > 420$ nm	100 mg catalyst, 8 mL ethanol, 72 mL H_2O, 25 ℃, N_2	131	1
Cu-TiO_2	300 W xenon lamp, AM 1.5	20 mg catalyst, 20 mL H_2O, 25ºC, N_2	79	2
Fe-TiO_2/Au	300 W xenon lamp, $\lambda > 420$ nm	100 mg catalyst, 80 mL H_2O, 25 ℃, N_2	50	3
Mo-$W_{18}O_{49}$	300 W xenon lamp, AM 1.5	10 mg catalyst, 10 mL H_2O (1 mM Na_2SO_3), 25 ℃, N_2	196	4
2.3% Mn-WO_3	300 W xenon lamp, AM 1.5	10 mg catalyst, 20 mL H_2O, 25 ℃, N_2	425	5
Au/$(BiO)_2CO_3$	300 W xenon lamp, AM 1.5	20 mg catalyst, 20 mL H_2O, 25 ℃, N_2	38	6
GSCe	6 W LED lamp	20 mg catalyst, 20 mL H_2O, 25 ℃, N_2	110	7
CdS:MoFe protein	405 nm diode, 25 mW·cm^{-2}	300 μL solution of 16.7 nM catalyst, 500 mM HEPES, 25ºC, N_2	19	8
Gd-IHEP-8	300 W xenon lamp, AM 1.5	20 mg catalyst, 100 mL H_2O, 25ºC, N_2	220	9
Au@UiO-66/PTFE membrane	300 W xenon lamp, $\lambda > 400$ nm	Membrane, 80 mL H_2O (0.5 M K_2SO_4), 25 ℃, N_2	360	10
5-FTNFs	300 W xenon lamp, AM 1.5	5 mg catalyst, 15 mL H_2O, 25 ℃, N_2	64	11
$AuRu_{0.31}$	300 W xenon lamp, AM 1.5	0.2 mg catalyst, 3 mL H_2O, 25 ℃, N_2	101	12

SUPPORTING REFERENCES

(1) Yang, J. H.; Guo, Y. Z.; Jiang, R. B.; Qin, F.; Zhang, H.; Lu, W. Z.; Wang, J. F.; Yu, J. C. High-Efficiency "Working-in-Tandem" Nitrogen Photofixation Achieved by Assembling Plasmonic Gold Nanocrystals on Ultrathin Titania Nanosheets. *J. Am. Chem. Soc.* **2018**, *140*,

8497–8508.

(2) Zhao, Y. X.; Zhao, Y. F.; Shi, R.; Wang, B.; Waterhouse, G. I. N.; Wu, L.-Z.; Tung, C.-H.; Zhang, T. R. Tuning Oxygen Vacancies in Ultrathin TiO_2 Nanosheets to Boost Photocatalytic Nitrogen Fixation up to 700 nm. *Adv. Mater.* **2019**, *31*, 1806482.

(3) Yang, J. H.; Bai, H. Y.; Guo, Y. Z.; Zhang, H.; Jiang, R. B.; Yang, B. C.; Wang, J. F.; Yu, J. C. Photodriven Disproportionation of Nitrogen and its Change to Reductive Nitrogen Photofixation. *Angew. Chem. Int. Ed.* **2021**, *60*, 927–936.

(4) Zhang, N.; Jalil, A.; Wu, D. X.; Chen, S. M.; Liu, Y. F.; Gao, C.; Ye, W.; Qi, Z. M.; Ju, H. X.; Wang, C. M.; Wu, X. J.; Song, L.; Zhu, J. F.; Xiong, Y. J. Refining Defect States in $W_{18}O_{49}$ by Mo Doping: A Strategy for Tuning N_2 Activation towards Solar-Driven Nitrogen Fixation. *J. Am. Chem. Soc.* **2018**, *140*, 9434–9443.

(5) Zhang, Y. D.; Hou, T. T.; Xu, Q.; Wang, Q. Y.; Bai, Y.; Yang, S. K.; Rao, D. W.; Wu, L. H.; Pan, H. B.; Chen, J. F.; Wang, G. M.; Zhu, J. F.; Yao, T.; Zheng, X. S. Dual-Metal Sites Boosting Polarization of Nitrogen Molecules for Efficient Nitrogen Photofixation. *Adv. Sci.* **2021**, *8*, 2100302.

(6) Xiao, C. L.; Hu, H.; Zhang, X. Y.; MacFarlane, D. R. Nanostructured Gold/Bismutite Hybrid Heterocatalysts for Plasmon-Enhanced Photosynthesis of Ammonia. *ACS Sustain. Chem. Eng.* **2017**, *5*, 10858–10863.

(7) Liu, S.; Teng, Z. Y.; Liu, H.; Wang, T. Y.; Wang, G. X.; Xu, Q.; Zhang, X. Y.; Jiang, M.; Wang, C. Y.; Huang, W.; Pang, H. A Ce-UiO-66 Metal–Organic Framework-Based Graphene-Embedded Photocatalyst with Controllable Activation for Solar Ammonia Fertilizer Production. *Angew. Chem. Int. Ed.* **2022**, *61*, e202207026.

(8) Brown, K. A.; Harris, D. F.; Wilker, M. B.; Rasmussen, A.; Khadka, N.; Hamby, H.; Keable, S.; Dukovic, G.; Peters, J. W.; Seefeldt, L. C.; King, P. W. Light-driven Dinitrogen Reduction Catalyzed by a CdS:Nitrogenase MoFe Protein Biohybrid. *Science* **2016**, *352*, 448–450.

(9) Hu, K. Q.; Qiu, P. X.; Zeng, L. W.; Hu, S. X.; Mei, L.; An, S. W.; Huang, Z. W.; Kong, X. H.; Lan, J. H.; Yu, J. P.; Zhang, Z. H.; Xu, Z. F.; Gibson, J. K.; Chai, Z. F.; Bu, Y. F.; Shi, W. Q. Solar-driven Nitrogen Fixation Catalyzed by Stable Radical-containing MOFs: Improved Efficiency Induced by a Structural Transformation. *Angew. Chem. Int. Ed.* **2020**, *59*, 20666–20671.

(10) Chen, L.-W.; Hao, Y.-C.; Guo, Y.; Zhang, Q.; Li, J.; Gao, W.-Y.; Ren, L.; Su, X.; Hu, L.; Zhang, N.; Li, S.; Feng, X.; Gu, L.; Zhang, Y.-W.; Yin, A.-X.; Wang, B. Metal–Organic Framework Membranes Encapsulating Gold Nanoparticles for Direct Plasmonic Photocatalytic Nitrogen Fixation. *J. Am. Chem. Soc.* **2021**, *143*, 5727–5736.

(11) Bo, Y. N.; Wang, H. Y.; Lin, Y. X.; Yang, T.; Ye, R.; Li, Y.; Hu, C. Y.; Du, P. Y.; Hu, Y. G.; Liu, Z.; Long, R.; Gao, C.; Ye, B. J.; Song, L.; Wu, X. J.; Xiong, Y. J. Altering Hydrogenation Pathways in Photocatalytic Nitrogen Fixation by Tuning Local Electronic Structure of Oxygen Vacancy with Dopant. *Angew. Chem. Int. Ed.* **2021**, *60*, 16085.

(12) Hu, C. Y.; Chen, X.; Jin, J. B.; Han, Y.; Chen, S. M.; Ju, H. X.; Cai, J.; Qiu, Y. R.; Gao, C.; Wang, C. M.; Qi, Z. M.; Long, R.; Song, L.; Liu, Z.; Xiong, Y. J. Surface Plasmon Enabling Nitrogen Fixation in Pure Water through a Dissociative Mechanism under Mild Conditions. *J. Am. Chem. Soc.* **2019**, *141*, 7807–7814.
